# Estimation of malaria haplotype and genotype frequencies: a statistical approach to overcome the challenge associated with multiclonal infections

**DOI:** 10.1186/1475-2875-13-102

**Published:** 2014-03-17

**Authors:** Aimee R Taylor, Jennifer A Flegg, Samuel L Nsobya, Adoke Yeka, Moses R Kamya, Philip J Rosenthal, Grant Dorsey, Carol H Sibley, Philippe J Guerin, Chris C Holmes

**Affiliations:** 1 WorldWide Antimalarial Resistance Network (WWARN), Oxford, UK; 2 Centre for Tropical Medicine, Nuffield Department of Clinical Medicine, University of Oxford, Oxford, UK; 3 Department of Statistics, University of Oxford, Oxford, UK; 4 Department of Medicine, Makerere University, Kampala, Uganda; 5 Uganda Malaria Surveillance Project, Kampala, Uganda; 6 Department of Medicine, University of California, San Francisco, USA; 7 Department of Genome Sciences, University of Washington, Seattle, WA, USA

**Keywords:** Frequency *versus* prevalence, Statistical model, Anti-malarial resistance

## Abstract

**Background:**

Reliable measures of anti-malarial resistance are crucial for malaria control. Resistance is typically a complex trait: multiple mutations in a single parasite (a haplotype or genotype) are necessary for elaboration of the resistant phenotype. The frequency of a genetic motif (proportion of parasite clones in the parasite population that carry a given allele, haplotype or genotype) is a useful measure of resistance. In areas of high endemicity, malaria patients generally harbour multiple parasite clones; they have multiplicities of infection (MOIs) greater than one. However, most standard experimental procedures only allow measurement of marker prevalence (proportion of patient blood samples that test positive for a given mutation or combination of mutations), not frequency. It is misleading to compare marker prevalence between sites that have different mean MOIs; frequencies are required instead.

**Methods:**

A Bayesian statistical model was developed to estimate *Plasmodium falciparum* genetic motif frequencies from prevalence data collected in the field. To assess model performance and computational speed, a detailed simulation study was implemented. Application of the model was tested using datasets from five sites in Uganda. The datasets included prevalence data on markers of resistance to sulphadoxine-pyrimethamine and an average MOI estimate for each study site.

**Results:**

The simulation study revealed that the genetic motif frequencies that were estimated using the model were more accurate and precise than conventional estimates based on direct counting. Importantly, the model did not require measurements of the MOI in each patient; it used the average MOI in the patient population. Furthermore, if a dataset included partially genotyped patient blood samples, the model imputed the data that were missing. Using the model and the Ugandan data, genotype frequencies were estimated and four biologically relevant genotypes were identified.

**Conclusions:**

The model allows fast, accurate, reliable estimation of the frequency of genetic motifs associated with resistance to anti-malarials using prevalence data collected from malaria patients. The model does not require per-patient MOI measurements and can easily analyse data from five markers. The model will be a valuable tool for monitoring markers of anti-malarial drug resistance, including markers of resistance to artemisinin derivatives and partner drugs.

## Background

The emergence and spread of resistance to anti-malarial drugs, such as chloroquine (CQ) and sulphadoxine-pyrimethamine (SP), have had a significant impact on public health [1,2]. Artemisinin-based combination therapy (ACT) is the first-line treatment for uncomplicated *Plasmodium falciparum* malaria in nearly all areas [3], however resistance to artemisinin derivatives, the key components of ACT, has been reported in Southeast Asia [4-8]. Since alternative treatments with equivalent tolerability and efficacy are currently unavailable [9], there is an urgent need to monitor emerging resistance to artemisinin derivatives and their partner drugs, including lumefantrine, amodiaquine, mefloquine, piperaquine, pyronaridine and sulphadoxine-pyrimethamine.

While *in vivo* studies are the “gold standard” for measuring the clinical efficacy of an anti-malarial drug, clinical treatment failure is a late marker of the spread of drug resistance and is complicated by host factors [10]. Genetic studies (see Table 1) can provide a complementary approach for monitoring drug-resistant parasites that is less complicated and expensive than *in vivo* studies [10]. In general, multiple genetic mutations in a single parasite are required for the elaboration of the resistant phenotype. The frequency of these genetic motifs (the proportion of parasites in the parasite population that carry a resistant allele, haplotype or genotype) is the metric needed to compare changes in resistance between studies conducted at different times, or in different sites [11]. However, estimation of frequencies is non-trivial because each patient may have multiple, genetically distinct malaria clones within their infection. In areas of high endemicity, it is common for the number of parasite clones in a blood sample from a single patient, the multiplicity of infection (MOI), to be more than one [12]. Based on the standard output of most current experimental methods, the prevalence of the markers considered (the proportion of individual patient blood samples that test positive for a given mutation or combination of mutations) is measured and is, therefore, commonly reported instead of frequency.

**Table 1:** Summary of terms

	
**Genetic study**	In a genetic study, *Plasmodium falciparum* DNA is extracted from infected human blood and genotyped to test for the presence of parasites that bear genetic markers of resistance (for a recent example see [13]).
**Genetic markers of resistance**	Markers of resistance are alleles in the parasite’s genome that have been associated with anti-malarial resistance either clinically or in the laboratory. The markers considered here are located at single nucleotide polymorphisms (SNPs) found within genes that encode anti-malarial drug targets. In the case of *Pfdhfr* and *Pfdhps*, markers of resistance are non-synonymous mutations, while sensitive markers are wild type alleles [14-16].
**Sulphadoxine-pyrimethamine**	Sulphadoxine-pyrimethamine (SP) is an anti-malarial drug comprising sulphadoxine and pyrimethamine. Both components act on the folate biosynthesis pathway: sulphadoxine inhibits dihydropteroate synthase, whereas pyrimethamine inhibits dihydrofolate reductase (reviewed in [17]). SP was widely used in the latter half of the 20th Century, and resistance to the drug is now extensive, particularly in Southeast Asia, and Eastern and Southern Africa (see [18] for a comprehensive review of antifolate resistance). Nonetheless, SP is still recommended for intermittent preventative treatment of malaria during infancy and pregnancy in many parts of sub-Saharan Africa, seasonal malaria chemoprevention (SMC) in combination with amodiaquine and as a partner drug of artemisinin derivatives in South Asia and the Horn of Africa [19,20].
**Haplotypes, genotypes and linkage phase**	Linkage phase describes the alignment of consecutive genetic markers on a chromosome. The resulting aligned set of multiple markers is called a haplotype. When markers are located in different genes and/or chromosomes, genotype defines the combination of haplotypes within a single parasite. *Plasmodium falciparum* is haploid throughout the human stage of its life cycle [21], so if a sample from a single patient is monoclonal, each of these genetic motifs can be clearly defined. However, it is often the case that a patient’s blood sample contains more than one clone. If these parasites differ with respect to two or more of the markers being examined, the molecular assay will show more than one marker at two or more positions (see Table 2). Then, it is not possible to define unambiguously the linkage phase, haplotype or genotype in a particular parasite.

In order to monitor trends in resistance across time and space, the metric of resistance must be defined on a common scale. Frequency provides such a metric, whereas prevalence does not. To illustrate this point, consider a hypothetical comparison between data collected from a village before and after the introduction of insecticide-treated bed nets (ITNs). Before the intervention, the average MOI was high. Since each patient was infected with several parasite clones, the resistant marker prevalence (the proportion of patient blood samples that tested positive for the resistant marker in pure form or mixed with the sensitive marker) was high, even though the resistant marker frequency (the proportion of parasite clones in the parasite population that carried the resistant marker) was low. Due to a drop in the average MOI, after the intervention most infections were monoclonal. Since each patient was now infected with a single clone, the prevalence of the resistant marker was equivalent to its frequency, which remained low. In other words, based on prevalence, the intervention appeared to be associated with a drop in resistance; however, the drop in prevalence was merely due to a drop in the average MOI (there had been no change in the frequency of the resistant marker in the parasite population). For this reason, it is not appropriate to compare prevalence estimates based on data collected under different average MOIs (such as two different sites, or one site at two different times); frequency estimates are required instead.

The previous example illustrates why prevalence should not be used to monitor spatial and/or temporal changes in resistance. However, prevalence estimates can also be misleading if they are used to approximate frequency at a single point in space and time because prevalence is liable to overestimate the frequency of common genetic motifs.

In practice, prevalence estimates are generated by scoring mixed SNPs (single nucleotide polymorphisms where both the sensitive and resistance markers were simultaneously detected) as either pure sensitive or pure resistant, depending on whether the prevalence of the sensitive or resistant marker is required. Additional counting methods to estimate gene frequency also exist [22,23]. These include discarding all discernibly multiclonal blood samples, which can lead to large losses of data; or discounting minority alleles at mixed SNPs. Due to the loss of valuable information regarding rare mutations, all of the conventional counting methods result in biased frequency estimates. For the reasons described below, they are also liable to lead to spurious haplotype and genotype reconstruction.

Since malaria parasites are haploid throughout the human stage of their life cycle [21], the haplotype (sequence of alleles within a gene) or genotype (if the alleles span multiple genes) of a single parasite clone in a monoclonal infection is clearly defined. When the MOI exceeds one, and the constituent clones differ at two or more examined SNPs, standard genotyping methods cannot determine which markers belong to which clone. This means that defining the linkage phase (see Table 1 for a definition), and therefore the haplotype or genotype, of each constituent clone is non-trivial. When haplotype or genotype reconstruction is performed using either prevalence estimates or frequency estimated based on conventional counting methods, the results can be misleading. For instance, discounting sensitive markers or minority markers at mixed SNPs can lead to spurious haplotype or genotype reconstruction (see Table 2).

**Table 2:** The analytical challenge presented by multiclonal infections: a hypothetical example of a multiclonal blood sample

**Hypothetical multiclonal infection**		**SNPs**				
		***Pfdhfr***			***Pfdhps***	
		**51**	**59**	**108**	**437**	**540**
**Unobserved true genotypes**	**Clone 1**	N	C	S	A	K
	**Clone 2**	N	C	S	A	K
	**Clone 3**	**I**	C	**N**	A	K
	**Clone 4**	N	**R**	**N**	A	K
	**Clone 5**	**I**	C	**N**	**G**	**E**
**Observed data**		N/**I**	C/**R**	S/**N**	A/**G**	K/**E**
**Example of an incorrect interpretation of the observed data**	**Genotype of clone inferred by discounting wild type markers**	**I**	**R**	**N**	**G**	**E**
	**Genotype of clone inferred by discounting minority markers**	N	C	**N**	A	K

Suppose a *P. falciparum* multiclonal blood sample was genotyped at single nucleotide polymorphisms (SNPs) in codons 51, 59 and 108 in *Pfdhfr* and codons 437 and 540 in *Pfdhps*. Markers are summarized by the amino acid residues they encode, which are denoted using the single letter amino acid code. Residues encoded by codons containing mutations are highlighted bold. The observed data are a direct consequence of the unobserved genotypes. Optimal genotyping sensitivity is assumed (all alleles are detected). Mutations were detected for all five codons genotyped; however, the interpretation that the infection contains the quintuple mutant is incorrect, since the linkage phase of the unobserved genotypes and the MOI are not captured in the observed data.

To address the problems associated with conventional counting methods and, therefore, harness the full potential of molecular methods for the surveillance of anti-malarial resistance, various statistical solutions have been proposed [11,24-27]. Statistical methods for genotype frequency estimation are desirable, since they take advantage of all the information in the data. For example, the freely available online program MalHaploFreq uses a multinomial distribution to model the unobserved genotypes in patient blood samples [11]. Maximum likelihood estimates of the genotype frequencies, which feature as probabilities in the multinomial distribution, are found using a hill-climbing algorithm. In fact, the majority of statistical methods use maximum likelihood estimation to generate point estimates of frequencies and accompanying confidence intervals [11,24,25,27]. Alternatively, a Bayesian framework, such as that developed by Wigger *et al.*[26], can be used to impute the unobserved variables, allow the propagation of uncertainty and enable the incorporation of specialist knowledge [28,29]. Since the aforementioned Bayesian model is reliant upon patient-level measurements of the MOI, an alternative Bayesian model, which is not reliant upon patient-level MOI measurements, was developed. The newly developed model, which uses prevalence data and a single estimate of the average MOI in the patient population to estimate genotype frequencies, is presented here.

## Methods

A statistical model was developed to estimate the frequency of *Plasmodium falciparum* genetic motifs (the proportion of parasite clones in the parasite population that carry a given allele, haplotype or genotype) using prevalence data. Henceforth, genotypes are referred to instead of genetic motifs; however, the same methods apply for alleles and haplotypes. In this section the model details are given; the methods of a simulation study, conducted to verify that the model provides accurate and precise frequency estimates, are summarized; and the application of the model to data collected from five sites in Uganda is described.

Given a dataset with N patients and s markers genotyped, the random variables of interest in the model were

1) the MOI for each of the i = 1 to N patient’s: **m** = m_i_,…, m_N_

2) the genotypes of the clones within the ith patient: G_ij_, j = 1, … , m_i_ , since the ith patient had m_i_ clones

3) for s binary (sensitive or resistant) markers, the genotype frequencies for the r=2^s^ possible genotypes: **π** = [π_1_, π_2_,…, π_r_]

The observed data for the ith patient were considered a direct consequence of the unobserved m_i_ genotypes (G_ij_, j = 1, … , m_i_) in each patient blood sample, assuming optimal detectability of minority alleles and negligible experimental error. See Table 2 for an example of how unobserved genotypes gave rise to the observed data.

Due to the Bayesian nature of the model, priors were specified for **m** and **π**. For the analyses of simulated data the prior for **m** was a Poisson distribution, left-truncated at one and right-truncated at eight, with parameter equal to a mean MOI of three, unless stated otherwise. The frequency **π** was assigned a uniform prior distribution, formally a Dirichlet prior with parameters set to one. Given **π** and the MOI for the ith patient (m_i_), the genotypes were assumed to be a realization from a multinomial distribution. The full mathematical details for the model are given in Additional file 1.

A Metropolis-Hastings Markov chain Monte Carlo (MCMC) sampling algorithm [28,29] was used to draw samples of genotype frequencies from the posterior distribution of the genotype frequencies conditional on the observed data, using recursive resampling to approximate the summation over the unknown MOIs and genotypes. Each time a new genotype was sampled within the recursive resampling scheme, the incomplete marker data, which sometimes arise due to unsuccessful genotyping outcomes or study design, were assigned a value and, therefore, imputed. The genotype frequency samples drawn using the MCMC algorithm were then used to infer the relevant frequencies. The median of the frequency sample set was used as a point estimate of the frequencies, and standard deviation as a measure of precision (low standard deviation corresponded to precise estimates). Accuracy was defined (for simulated datasets only) as the absolute difference between the known (simulated) frequency and the point estimated from the sample set generated by the model, averaged over the point estimates for the different genotypes compatible with a given dataset (low values indicated accurate estimates). The model was written and implemented in R [30], on a 64-bit computer with 16.0 Gb of random access memory and an Intel(R) Core(TM) i7-2600 central processing unit (CPU) @ 3.40GHz processor.

Model performance was assessed using a series of simulated datasets (see Additional file 2 for details). To assess MCMC convergence, the within and between sequence variance of parallel chains were compared. The speed, precision and accuracy were reported for datasets, as the size of the dataset (50, 100 and 1,000 blood samples) and the number of markers (one to five) varied. The sensitivity of the frequency estimates to key model assumptions, including optimal detectability, the prior MOI distribution and its parameter, and the effect of discarding blood samples with incomplete data, were also assessed.

**Figure 1:**
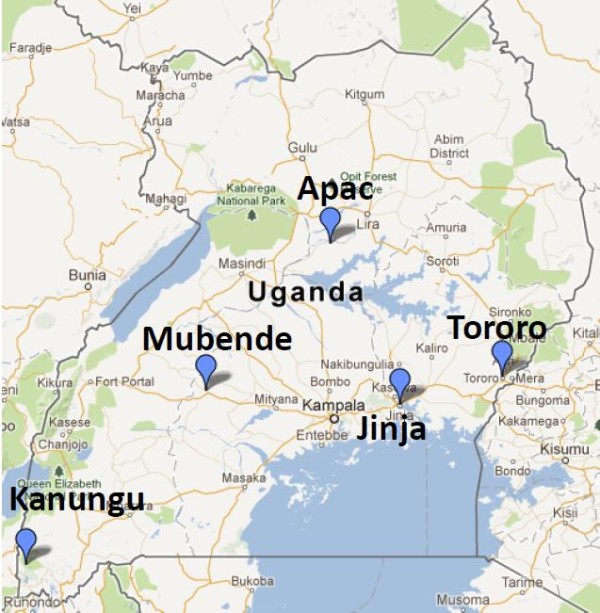
**Study sites of the Ugandan field data.** The numbers of patient blood samples analysed from each site were 358 (Kanungu), 354 (Mubende), 333 (Jinja), 334 (Tororo), and 359 (Apac). The blood samples were collected between December 2002 and May 2004 (see [31] for more details). The mean MOI reported for each site was 2.64 (Kanungu), 3.01 (Mubende), 2.62 (Jinja), 4.46 (Tororo) and 4.16 (Apac).

**Figure 2:**
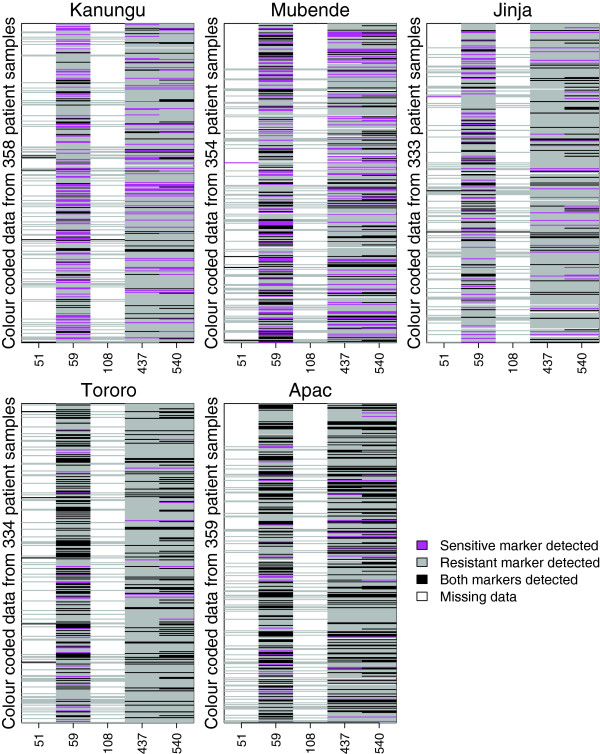
**Visualisation of raw data.** Prevalence data are colour coded: pink, detection of the sensitive marker only; grey, detection of the resistant marker only; black, detection of both sensitive and resistant markers (mixed SNP); white, missing datum. Rows differentiate the data derived from different patient blood samples; columns differentiate the data for each of the markers genotyped in codons 51, 59, 108 (in *Pfdhfr*) and 437 and 540 (in *Pfdhps*).

To demonstrate the ability of the model to estimate genotype frequencies for real patient data, the model was applied to data from a multisite drug efficacy trial. From five study sites in Uganda (Figure 1), prevalence data for markers at codons 51, 59, 108 in the *Plasmodium falciparum* gene encoding dihydrofolate reductase (*Pfdhfr*) and 437 and 540 in the *Plasmodium falciparum* gene encoding dihydropteroate synthase (*Pfdhps*), which are associated with SP resistance [18], were collected between December 2002 and May 2004 (see [31] for further details). All patient blood samples were genotyped at codons *Pfdhfr* 59, *Pfdhps* 437 and *Pfdhps* 540, while 80 blood samples from each site were randomly selected for genotyping at codons *Pfdhfr* 51 and *Pfdhfr* 108. The structure of the missing data is apparent in the visualisation of the raw data (Figure 2).

Due to interactions between mutant sites, not all genotypes are biologically viable [32]. Furthermore, due to the spread of a limited number of *Pfdhfr* mutant haplotype linages [33], preceding the emergence and spread of a limited number of *Pfdhps* mutant haplotype linages [34], *Pfdhfr* mutations almost always accompany *Pfdhps* mutations, and SP resistant genotypes vary geographically [35]. In terms of the statistical model, it was therefore important to allow for dependence within and between *Pfdhfr* and *Pfdhps* by running the model on the *Pfdhfr* and *Pfdhps* data combined. Due to the Bayesian framework, a subjective Dirichlet prior could have been used to incorporate information about the viability of the different genotypes. However, an objective, uniform prior was specified, meaning that all theoretically possible genotypes were regarded *a priori* as biologically feasible and equally probable. Doing so provides an objective basis against which the validity of the results can be compared. For the analyses of field data, the prior for **m** was a geometric distribution, left-truncated at one and right-truncated at eight. The parameter of the truncated geometric distribution was set equal to the reciprocal of the mean MOI estimate at the relevant site (MOI estimates were based on external information provided by the investigators [31]; see the legend of Figure 1, or Table 3 or 4 for mean MOI values. To test the sensitivity of the frequency estimates to the prior distribution parameter, the algorithm was rerun for MOI estimates at the upper and lower confidence interval limits (derived using a t-distribution and the reported sample standard deviation) of the mean MOI estimate. Since the true genotype frequencies were unknown, to gain insight into the accuracy of the estimates, a replicate analysis using simulated data based on the field data was performed (see Section 3 of Additional file 2 for details). This approach allows comparison between the true and estimated frequencies for data that mimic the field data.

**Table 3:** Ugandan field data characteristics stratified by site

**Characteristic**	**Study site**				
	**Jinja**	**Kanungu**	**Mubende**	**Apac**	**Tororo**
No. of patient blood samples	333	358	354	359	334
No. completely missing data (%)	5 (2)	2 (1)	9 (3)	4 (1)	0 (0)
No. partially missing data (%)	249 (75)	276 (77)	266 (75)	275 (77)	254 (76)
No. discernably multiclonal^†^ (%)	83 (25)	75 (21)	130 (37)	182 (51)	149 (45)
Reported mean MOI	2.62	2.64	3.01	4.16	4.46

†A blood sample was considered discernibly multiclonal if it had one or more mixed SNPs (SNPs where the sensitive and resistance markers were simultaneously detected).

**Table 4:** The prevalence of blood samples that test positive for all five mutant markers in pure or mixed form, and frequency of the quintuple mutant genotype, stratified by site

	**Jinja**	**Kanungu**	**Mubende**	**Apac**	**Tororo**
Reported Mean MOI	2.62	2.64	3.01	4.16	4.46
Marker prevalence	0.77	0.62	0.61	0.89	0.91
(**I, R, N, G** and **E**)	(0.72-0.81)	(0.57-0.67)	(0.56-0.66)	(0.85-0.92)	(0.87-0.93)
Genotype frequency (**IRNGE**)	0.65	0.54	0.46	0.65	0.70
	(0.60-0.70)	(0.49-0.59)	(0.40-0.51)	(0.60-0.69)	(0.66-0.74)

The reported mean MOI was extracted from the literature [31]. For each site, the prevalence of patient bloods samples that test positive for all five mutant markers (**I, R, N, G,** and **E**) in pure or mixed form was calculated by scoring mixed SNPs as mutant, then calculating the proportion of patient samples with mutant markers at each of the five SNPs (95% confidence intervals in parentheses). The frequency of the quintuple mutant genotype (**IRNGE**) was estimated by the model (95% credible intervals in parentheses).

## Results

### Model performance based on simulated data

As outlined in Methods, the model was tested on both simulated data and data collected from five sites in Uganda. In this section, the results from the simulation study, designed to test model performance, are summarized (see Sections 1 and 2 of Additional file 2 for more details). Convergence of the sampling algorithm was rapid: 50,000 iterations were sufficient. The results of the algorithm were robust: frequency estimates varied by less than 0.02 when the algorithm was started from different initial genotype frequencies. The algorithm was computationally fast, typically taking less than 10 min to analyse a dataset comprising 100 blood samples and five markers. Importantly, the precision and accuracy of the statistical estimates increased with the number of blood samples analysed by the model. Notably, the accuracy of the estimates generated by the statistical model was superior to those obtained when a commonly employed counting method was applied to the same datasets. For datasets with partial data, imputation of incomplete data afforded favourable accuracy and precision (see Additional file 1 for details of the how imputation was implemented). The sensitivity of the experimental methods to detect minority markers in multiclonal patient blood samples was also an important parameter to consider (the incorporation of this parameter into future extensions of the model is of high priority). The current model, however, appeared to be robust to 90% detectability (Section 2, Additional file 2). Not surprisingly, when detectability dropped to 70%, accuracy decreased. Running this model on datasets generated for three or fewer SNPs given a clonal detectability less than 90% is therefore not advised. As expected, to a small extent, the model was sensitive to MOI prior mis-specification. The principal purpose of the model was to estimate genetic motif frequencies (the proportion of parasite clones in the parasite population that carry a given allele, haplotype or genotype), it was not designed to estimate patient-level MOIs. Providing the model is used for its primary purpose, MOI prior mis-specification is not problematic. However, if patient-level MOI estimates are required, a thorough sensitivity check is possible. In any case, selection of a prior distribution that best fits the field data and repeat analysis within a reasonable range of the reported MOI is recommended. If the average MOI is unknown, a uniform MOI, which does not require MOI specification, can be used.

### Results from the field data

Having established that the model works on simulated data, its utility was assessed on field data from five sites in Uganda that have variable levels of malaria transmission. Table 3 shows that at each of the five sites, at least 20% of the patient blood samples were multiclonal (the detection of one or more mixed SNPs was indicative of a multiclonal infection), while in Apac, where the endemicity was reportedly very high, more than half the blood samples were multiclonal. Usually in this kind of analysis some assays do not yield data and must be treated as missing. In these field sites, it was known that the resistance markers at codons 51 and 108 were virtually fixed in the parasite population [31], so those were tested in only 80 blood samples. This allowed an excellent demonstration of the capacity of the model to deal with incomplete data using imputation.

The genotype frequencies (proportion of parasite clones in the parasite population that carry a given genotype) estimated by the model using a geometric prior over the MOI are reported in Table 5. The SNPs at three codon positions in *Pfdhfr* (N51**I**, C59**R** and S108**N**) and two codons in *Pfdhps* (A347**G** and K540**E**) were assessed. The model estimated frequencies for each theoretically possible genotype; however, only those with frequency greater than 0.03 at one or more of the sampling sites are reported. The frequency of the sensitive, wild type (NCSAK) genotype was negligible at each of the sites while the highly resistant, quintuple mutant (**IRNGE**) had the highest estimated frequency. The frequencies of the quadruple (**I**C**NGE)**, triple (**IRN**AK), and double mutant (**I**C**N**AK) exceeded 0.03 in at least one site.

**Table 5:** **Estimated *Plasmodium falciparum *genotype frequencies, and their values at the extremes of their 95**% **credible intervals (in parentheses), in the five Ugandan study sites**

**Site**	**Genotype frequencies**			
	**ICNAK**	**IRNAK**	**ICNGE**	**IRNGE**
Tororo	0.02 (0.01-0.03)	0.02 (0.01-0.04)	0.13 (0.10-0.17)	0.70 (0.66-0.74)
Jinja	0.03 (0.01-0.05)	0.04 (0.02-0.06)	0.13 (0.10-0.17)	0.65 (0.60-0.70)
Apac	0.05 (0.03-0.08)	0.07 (0.05-0.10)	0.11 (0.08-0.14)	0.65 (0.60-0.69
Kanungu	0.06 (0.03-0.08)	0.05 (0.03-0.08)	0.17 (0.13-0.21)	0.54 (0.49-0.59)
Mubende	0.07 (0.05-0.10)	0.10 (0.07-0.13)	0.20 (0.16-0.24)	0.46 (0.40-0.51)

Frequencies were estimated using the model, the prevalence data and the mean MOI reported by Francis *et al.* at each site [31]. The genotypes are defined by the amino acid residues they encode at codons 51, 59, 108 in *Pfdhfr*, and 437 and 540 in *Pfdhps*. The amino acid residues are given by their one letter amino acid code. Residues encoded by resistance markers are underlined. All genotypes that are theoretically compatible with the data were considered by the model, however, only those with a frequency greater than 0.03 at one or more sites are reported.

At each site there appeared to be an inverse relationship between the frequency of the quintuple mutant (**IRNGE**) and that of the remaining genotypes. Given the expected selective advantage of the quintuple mutant under SP drug pressure, this relationship may reflect the displacement of clones carrying the less resistant genotypes by those carrying the highly resistant genotype. It is particularly important that the model enabled precise estimation of the frequency of this highly resistant genotype (**IRNGE**) across sites that differ in MOI, since it has been shown to be highly predictive of clinical failure of SP [36], and is, therefore, an important genotype for molecular surveillance of SP resistance.

### Comparison of estimates from the statistical model and conventional counting methods

Most conventional counting methods can only measure the prevalence of markers (how many of the patient blood samples test positive for each marker type of interest, either alone or mixed with the alternative type), not their frequency. Using prevalence data, various counting strategies have been used to estimate the frequency of the marker in the parasite population [22,23]. To assess these approaches, the frequency estimates from the statistical model were compared with those from commonly used counting methods using the data from Uganda (see Section 3, Additional file 2 for full details).

Counting methods are all based on a simple idea: using a set of assumptions, construct a subsample of the dataset that contains no multiclonal patient blood samples or samples with missing data, such that frequencies can be directly calculated using proportions. Three different approaches are compared: discarding any blood samples with missing data and/or evidence of multiclonality; assuming all of the markers at codons 51 and 108 were mutant, and then discarding any remaining blood samples with missing data and/or evidence of multiclonality; assuming all mixed SNPs were purely resistant (this approach generates prevalence estimates for the proportion blood samples that show evidence of all five mutant type markers, **I**, **R**, **N**, **G** and **E** in either pure or mixed form), and then discarding any remaining blood samples with missing data. Confidence intervals surrounding counting method estimates were based on the Wilson score interval. Genotypes that were not represented in the subsample were assumed to have zero frequency. Each approach was applied to a field sample set, and the frequency estimates compared with the statistical model estimation.

Point estimates from the three different counting methods differed greatly compared with the point estimates generated by the model using different MOI distributions (see Section 3, Additional file 2). Moreover, the credible intervals (Bayesian equivalent of confidence intervals) surrounding the estimates generated by the model were far smaller than the confidence intervals resulting from the counting methods. This outcome is not surprising, since the various counting methods produced subsets of the data that necessarily varied in size. This demonstrated the increased precision of the model output, another important asset of the model approach. Overall, each of the counting methods excluded information from a considerable portion of each dataset, and these smaller subsets of the data produced the outputs that were far less consistent with each other and with the output from the model.

The accuracy of the estimates generated by the model using the field data could not be compared with those generated using counting methods, since the true genotype frequencies of the data from the field are, of course, unknown. However, it was possible to compare the accuracy of the statistical estimates of the simulated data, which were simulated under the model using the genotype frequencies from these field data. When that was done, the accuracy of the model output was superior to that of the counting estimates (see Section 3 Additional file 2).

For comparison, the prevalence of patient blood samples that show evidence of all five mutant type markers, **I**, **R**, **N**, **G** and **E** in either pure or mixed form at each of the five sites is reported in Table 4. It is important to note that an infection that tests positive for all five mutant markers does not necessarily contain the quintuple mutant genotype (consider Table 2 for example). The prevalence of blood samples that text positive for all five mutant markers is liable to overestimate the frequency of the quintuple mutant genotype. The degree of overestimation depends on the mean MOI: given a fixed frequency, prevalence is expected to increase as the mean MOI increases. Based on the estimates generated by the model, it is difficult to comment on the expected relationship between the mean MOI and prevalence across all five sites because the estimated frequency of the quintuple mutant genotype and the mean MOI varied from site to site. Jinja and Apac, however, do have equivalent frequency estimates, and as expected, the largest discrepancy between prevalence and frequency between these two sites is in Apac, the site with the greater mean MOI (Table 4).

## Discussion

The frequency of a genetic motif (the proportion of parasite clones in the parasite population that carry a given marker, haplotype or genotype) of anti-malarial resistance in a parasite population is an extremely useful measure of anti-malarial resistance. Unfortunately, what can be measured is the prevalence of markers, the proportion of patient blood samples that test positive for a single marker or combination of markers. This parameter depends strongly on the number of different clones in the sample, the MOI, and does not capture the linkage phase of the haplotypes or genotypes, which are the key determinants of the resistance phenotype. In the recent decade, there has been considerable progress in malaria control in many regions, manifest in lower malaria transmission and the concomitant reduction in the mean MOI. Comparisons of the changes in frequency over time or from different sampling sites are now more important than ever. Various estimates of the frequency based on direct counting of the prevalence data have been used, but most approaches require discarding valuable information, and may also lead to biased estimates.

In contrast, the model uses all available information to infer genotypes for the clones within each individual infection. It therefore reconstructs haplotypes and genotypes, provides a consistent method of frequency estimation, and avoids the loss of information that results from the adjustments made for multiclonal patient blood samples and unsuccessful genotyping outcomes. Application of the model to field data will allow the changes in genetic motif frequencies to be tracked, yielding important information on the dynamics of resistance.

Like any model, however, it is important to note that a number of assumptions have been made to construct the model. To assess the impact of various assumptions, a simple sensitivity analysis was performed using simulated data. The model was robust to changes in the initial frequency estimates and to the assumption that minority alleles are detected, providing the limit of detectability is 90% or more, but sensitive to deviations in the mean MOI and the distribution used to model the mean MOI. In light of these results, the use of this model for data generated using an assay that has a limit of detectability less than 90% is not recommended. An investigation to find the MOI distribution that provides the best fit to the data is recommended, followed by repeat analyses of the data, each time varying the mean MOI value within a reasonable range (such as the limits of the 95% confidence interval of the mean MOI), to establish the sensitivity of the results to its value.

Several key differences set this model apart from existing methods of malaria haplotype and genotype frequency estimation [11,24-27]. First, in contrast to all previously published methods, the model makes use of all available data, including those that are incomplete due to unsuccessful genotyping outcomes or study design. Second, in contrast to the recently published Bayesian method [26] and the model underpinning the freely available online software MalHaploFreq [11], this model is not reliant upon per-patient measurements of the MOI. Third, in contrast to some existing approaches [11,24,27], it enables rapid analysis of data from three or more markers. Superior assumptions regarding detectability and experimental error are incorporated into alternative models [11,26]. It is especially important to take into account the suboptimal detectability of minority clones, which was addressed by Hastings and colleagues using an indicator function [11,22], when the data are derived from polymerase chain reaction (PCR) methods and individual per-patient MOI measurements are regarded as fixed. However, the latter is not the case in the current model (patient-level MOIs are treated as unobserved random variables).

Model development was primarily motivated by a desire to make use of all available prevalence data to estimate frequencies, including prevalence data for which patient-level MOI measurements did not exist. It was not designed to estimate patient-level MOIs, and so does not obviate the need for MOI measurements in general. Adaptation of the model, to allow the incorporation of patient-level MOI estimates, is an important consideration for the future. However, in contrast to existing methods that regard the MOI as a fixed quantity [11,26], any extensions of the current model would preserve the current treatment of MOIs as random variables, perhaps using patient-level MOI measurements to inform patient level MOI distributions, allowing patient-level variation in the MOI.

## Conclusion

Genetic monitoring of *Plasmodium falciparum* plays an important role in the timely surveillance of anti-malarial drug resistance. The utility of a Bayesian model, designed to estimate *Plasmodium falciparum* genetic motif frequencies (proportions of parasite clones in the parasite population that carry given alleles, haplotypes or genotypes) has been demonstrated using data on markers of SP resistance. Its applicability, however, extends beyond SP to markers of resistance to lumefantrine, amodiaquine, mefloquine, piperaquine, pyronaridine. Moreover, the recent identification of molecular markers of artemisinin resistance in Cambodia [37], opens the possibility for using this model to compare the frequency of these markers in isolates from different sites or times. This is the first model that combines rapid analysis of three or more SNPs, using all available data (including those that are incomplete due to unsuccessful genotyping outcomes), without reliance on measurements of the MOI in individual patient blood samples. In the past, large amounts of valuable data have either been discarded or not used to their full capacity. It is imperative that a similar scenario is averted before widespread surveillance of resistance to artemisinins. The development of an accurate, consistent method for deriving comparable estimates of *Plasmodium falciparum* genotype frequencies, using known markers of multigenic resistance, provides a means to harness the full potential of current and prospective markers of anti-malarial resistance.

## Abbreviations

WWARN: WorldWide Antimalarial Resistance Network; MOI: Multiplicity of infection; CQ: Cloroquine; SP: sulphadoxine-pyrimethamine; ACT: Artemisinin-based combination therapy; ITNs: Insecticide-treated nets; SNPs: Single nucleotide polymorphism; MCMC: Markov chain Monte Carlo; Pfdhfr: *Plasmodium falciparum* gene encoding dihyrofolate reductase; Pfdhps: *Plasmodium falciparum* gene encoding dihydropteraote synthase; PCR: Polymerase chain reaction.

## Competing interests

The authors declare that they have no competing interests.

## Authors’ contributions

CH, JF, AT conceived the model. AT wrote, implemented and tested the algorithm, and drafted the manuscript. PG, CHS, JF, CH, and PR edited the manuscript. PR, MK, SN, AY, and GD contributed data and participated in the editing. All authors read and approved the final manuscript.

## Supplementary Material

Additional file 1 Mathematical description of the model, including a graphical visualisation of the model quantities and their conditional dependencies. Click here for file

Additional file 2 Full details of the simulation studies and analyses of the field data. Click here for file
