# Supplementary material for: Estimation of malaria haplotype and genotype frequencies: a statistical approach to overcome the challenge associated with multiclonal infections
Source: Malar J. 2014 Mar 17;13:102. doi: 10.1186/1475-2875-13-102 (PMC4004158; doi:10.1186/1475-2875-13-102)
Supplement: Additional file 1 — Mathematical description of the model, including a graphical visualisation of the model quantities and their conditional dependencies. [file 1475-2875-13-102-S1.pdf]

Additional file 1

1 Mathematical details of the model

1.1 Notation

Suppose n *Plasmodium falciparum* positive patient blood samples have been genotyped at s single nucleotide polymorphisms (SNPs). The observed data are a consequence of the alleles (if only one SNP is genotyped), haplotypes (if multiple SNPs in the same gene are genotyped), or genotypes (if multiple SNPs beyond a single gene are genotyped) of the *Plasmodium falciparum* clones that reside in the blood samples (for example, see Table A1.1). The observed data are stored in the n by s matrix $\boldsymbol{Y}$, so that the ith row corresponds to the ith blood sample. A single allele is henceforth referred to as a marker. Markers are categorised as either sensitive or resistant[1]. When the ith blood sample is genotyped at the jth SNP, the observed datum, y_{ij}, denotes the detection of purely sensitive or purely resistant markers ('0' or '1' respectively); or the simultaneous detection of both sensitive and resistance markers ('0.5'). SNPs at which the sensitive and resistance markers are simultaneously detected are henceforth referred to as mixed. '99' represents a missing datum. For example, $\boldsymbol{y}_i = (0.5, 99, 0)$ denotes the detection of both sensitive and resistant markers at SNP one of the ith blood sample, a missing datum at SNP two, and two or more sensitive markers at SNP three[2]. The assumption that missing data are missing at random and that the missing mechanism is ignorable is made[3]. Furthermore, 100% detectability of minority markers is assumed, and experimental error is considered negligible[4].

[1]Multiallelic SNPs are compatible with the model providing that each allele can be categorised as either a sensitive or a resistant marker.

[2]Since SNP one is mixed, the ith blood sample is discernibly multiclonal. The '0' detected at SNP three must, therefore, represent two or more sensitive markers.

[3]The term 'ignorable' has a technical definition [Little RJA, Rubin DB: Statistical Analysis with Missing Data. 2nd edition. Wiley-Blackwell, 2002]. The missing data mechanism is ignorable because the missing data are missing at random (the probability that a datum is missing does not depend on its value) and the parameters governing the missing mechanism, (for example, DNA quantity) are not related to the parameters of interest (the frequencies of the genetic motifs).

[4]'Since all models are wrong the scientist must be alert to what is importantly wrong' [Box GEP: Science and Statistics. J. Am. Stat. Assoc. 1976, 71:791799]. Heeding Box's advice, a thorough investigation was performed to ascertain the sensitivity of the model to the simplifying assumptions (see Additional File 2).

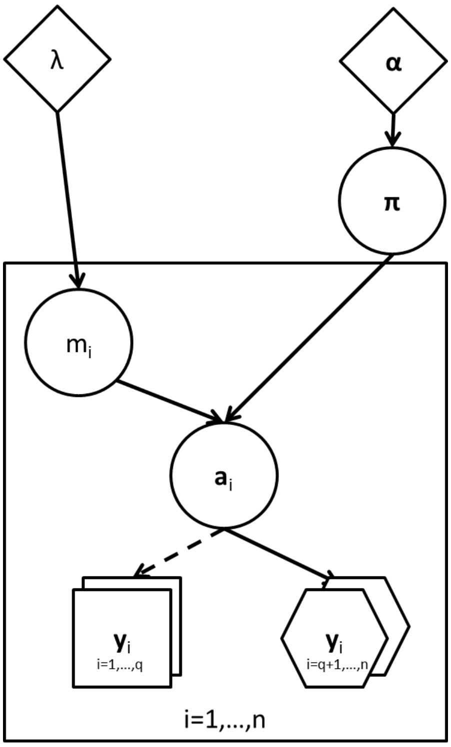

Figure A1.1: A directed acyclic graph representing the model quantities and their conditional dependencies. Solid arrows represent probabilistic dependencies, whereas the dashed arrow represents a deterministic dependency. Circles denote unknown quantities. The squares represents the data per patient blood sample, $\boldsymbol{y}_i$, where $i = 1, \cdots, q$ include no missing data. The hexagons denote the data per blood samples, $\boldsymbol{y}_i$, where $i = q+1, \cdots, n$ are incomplete (unsuccessful genotyping outcome at one or more SNPs). The diamonds represent the hyperparameters of the priors. The repetitive structure of the n blood samples within the dataset is represented by the stacked rectangles and hexagons. The joint distribution of the data and random quantities is $Pr(\boldsymbol{Y}, \boldsymbol{A}, \boldsymbol{\pi}, \boldsymbol{m}) = Pr(\boldsymbol{Y}|\boldsymbol{A})\, Pr(\boldsymbol{A}|\boldsymbol{m}, \boldsymbol{\pi})\, Pr(\boldsymbol{m})\, Pr(\boldsymbol{\pi})$.

Suppose $r \leq 2^s$ genotypes are compatible with the observed data, $\boldsymbol{Y}$. Let the n by r matrix $\boldsymbol{A}$ be the matrix in which the unobserved patient genotype counts are stored[5]. The genotype counts represent the numbers of clones characterised by each of the 1 to r genotypes in each of the n blood samples. The total genotype count per blood sample is equal to the total number of clones present in the blood sample, which is the multiplicity of infection (MOI). Let $\boldsymbol{m} = (m_1, \cdots, m_n)$ denote the MOIs for the n blood samples, so the sum of the ith row of $\boldsymbol{A}$ is m_i (in other words $\sum_{i=1}^{n} \boldsymbol{a}_i = m_i$). Akin to the genotype counts, they are unobserved. Let $\boldsymbol{\pi} = (\pi_1, \cdots, \pi_r)$ denote the vector of genotype frequencies. $\pi_1, \cdots, \pi_r$ are estimates of the clonal genotype frequencies (the proportions of parasite clones in the *Plasmodium falciparum* population that carry genotypes 1 to r). The model quantities and their conditional dependencies are depicted in Figure A1.1. A list of notation is included in Section 1.2. A hypothetical example, complete with unobserved variables and mathematical notation, is included in Section 2.

[5]A second matrix, $\boldsymbol{H}$, in which genotype counts are also stored, is introduced in Sections 1.2 and 3. $\boldsymbol{H}$ is required for model specification (Section 3), whereas $\boldsymbol{A}$ is used in the sampling algorithm (Section 3.1). Since $\boldsymbol{A}$ is more intuitive (it is a matrix in which patient genotype counts are stored), it is used to introduce the model.

1.2 List of notation

n number of blood samples in the dataset.

s number of SNPs genotyped.

r number of genotypes compatible with the observed data ($r \leq 2^s$).

$\boldsymbol{Y}$ n by s data matrix: $\boldsymbol{Y} = (\boldsymbol{y}_1, \cdots, \boldsymbol{y}_n)^T$, where $\boldsymbol{y}_i = (y_1, \cdots, y_s)^T$ and $y_{ij} \in \{0, 1, 0.5, 99\}$ $\forall$ $i = 1, \cdots, n$, and $j = 1, \cdots, s$.

m_{max} maximum MOI, set by the user (equal to eight in the current model).

$\boldsymbol{m}$ vector of n MOIs: $\boldsymbol{m} = (m_1, \cdots, m_n)$, where $m_i \in \{1, \cdots, m_{max}\}$ $\forall$ $i = 1, \cdots, n$ and $m_i = \sum_{j=1}^{r} a_{ij}$ (a_{ij} is defined below).

$\boldsymbol{A}$ n by r matrix of genotype counts: $\boldsymbol{A} = (\boldsymbol{a}_1, \cdots, \boldsymbol{a}_n)^T$, where $\boldsymbol{a}_i = (a_1, \cdots, a_r)^T$ and $a_{ij} \in \{0, \cdots, m_{max}\}$ $\forall$ $i = 1, \cdots, n$ and $j = 1, \cdots, r$.

$\boldsymbol{\pi}$ genotype frequencies, $\boldsymbol{\pi} = (\pi_1, \cdots, \pi_r)$, where $\pi_i \in [0, 1]$ $\forall$ $i = 1, \cdots, r$ and $\sum_{i=1}^{r} \pi_i = 1$.

k number of theoretically possible vectors of genotype counts given a specified MOI, m: $k = \binom{m+r-1}{r-1}$, given that there are r genotypes.

$\boldsymbol{H}$ k by r matrix of theoretically possible genotype counts given a specified MOI, m. $\boldsymbol{H} = (\boldsymbol{h}_1, \cdots, \boldsymbol{h}_k)^T$, where $\boldsymbol{h}_g = (h_1, \cdots, h_r)^T$, $h_{gj} \in \{0, \cdots, m\}$ $\forall$ $g = 1, \cdots, k$, $j = 1, \cdots, r$. The matrix $\boldsymbol{H}$ is similar to $\boldsymbol{A}$, in that it is a matrix of genotype counts; however, $\boldsymbol{H}$ contains all possible genotype counts given m, whereas $\boldsymbol{A}$ contains the genotype counts for the n blood samples in the dataset. For example, suppose two SNPs are genotyped, such that $r = 4$, since there are four possible genotypes (00, 10, 01, 11), and $m = 2$, then

$$\boldsymbol{H} = \begin{pmatrix} 2 & 0 & 0 & 0 \\ 0 & 2 & 0 & 0 \\ 0 & 0 & 2 & 0 \\ 0 & 0 & 0 & 2 \\ 1 & 1 & 0 & 0 \\ 1 & 0 & 1 & 0 \\ 1 & 0 & 0 & 1 \\ 0 & 1 & 1 & 0 \\ 0 & 1 & 0 & 1 \\ 0 & 0 & 1 & 1 \end{pmatrix}.$$

Distinct MOI values yield different $\boldsymbol{H}$ matrices because the number of theoretically possible combinations of genotype counts, k, is a function of the specified MOI, $k = f(m)$ (see above).

$\boldsymbol{\alpha}$ hyperparameter on the prior of $\boldsymbol{\pi}$: $\boldsymbol{\alpha} = (\alpha_1, \cdots, \alpha_r)$. $\boldsymbol{\alpha}$ is the parameter vector of a Dirichlet distribution (see Section 3).

λ hyperparameter on the prior of $\boldsymbol{m}$ (see Section 3).

2 Hypothetical example

	Observed data			Unobserved	
Blood sample	SNP 1	SNP 2	SNP 3	MOI	Genotype counts†
Patient 1	0	0	0	1	(1, 0, 0, 0, 0, 0, 0, 0)
Patient 2	0	1	0	4	(0, 0, 4, 0, 0, 0, 0, 0)
Patient 3	1	1	1	3	(0, 0, 0, 0, 0, 0, 0, 3)
Patient 4	0.5	0.5	0.5	2	(1, 0, 0, 0, 0, 0, 0, 1)
Patient 5	0	0.5	99	3	(1, 0, 1, 1, 0, 0, 0, 0)

Table A1.1: A hypothetical dataset based on five malaria patients. The observed data are shown on the left of the vertical division. For a given SNP, '0' denotes the detection of sensitive markers only, '1' denotes the detection of resistant markers only, '0.5' denotes the simultaneous detection of both sensitive and resistant markers and '99' denotes a missing datum (due to a failed genotyping outcome, for example).The genotype counts and MOIs give rise to the observed data, but they themselves are unobserved, and are hence shown on the right of the vertical division. †Genotype counts are represented by $(n_{(000)}, n_{(100)}, n_{(010)}, n_{(001)}, n_{(110)}, n_{(101)}, n_{(011)}, n_{(111)})$, where $n_{(000)}$ is the number of clones with genotype 000. For a given genotype '0' denotes a single sensitive marker and '1' denotes a single resistant marker. For example, the genotype count (1, 0, 0, 0, 0, 0, 0, 1) indicates that the fourth patient blood sample contains two clones, one with genotype 000 and one other with genotype 111.

Suppose five hypothetical blood samples are genotyped at three SNPs (Table A1.1). In accordance with the model notation introduced above, the data are stored in the $n \times s$ (5×3) matrix $\boldsymbol{Y}$; the unobserved genotype counts are stored in the $n \times r$ (5×8) matrix $\boldsymbol{A}$; and the MOIs are stored in the vector $\boldsymbol{m}$:

$$\boldsymbol{Y} = \begin{pmatrix} 0 & 0 & 0 \\ 0 & 1 & 0 \\ 1 & 1 & 1 \\ 0.5 & 0.5 & 0.5 \\ 0 & 0.5 & 99 \end{pmatrix}, \ \boldsymbol{A} = \begin{pmatrix} 1 & 0 & 0 & 0 & 0 & 0 & 0 & 0 \\ 0 & 0 & 4 & 0 & 0 & 0 & 0 & 0 \\ 0 & 0 & 0 & 0 & 0 & 0 & 0 & 3 \\ 1 & 0 & 0 & 0 & 0 & 0 & 0 & 1 \\ 1 & 0 & 1 & 1 & 0 & 0 & 0 & 0 \end{pmatrix}, \ \boldsymbol{m} = \begin{pmatrix} 1 \\ 4 \\ 3 \\ 2 \\ 3 \end{pmatrix}$$

Since the genotype counts and MOIs are known (because this is a hypothetical example), the sample genotype frequencies are directly calculable as the proportion of each

genotype in the dataset. In other words,

$$\begin{aligned}
\boldsymbol{\pi} &= \left(\pi_{(000)}, \pi_{(100)}, \pi_{(010)}, \pi_{(001)}, \pi_{(110)}, \pi_{(101)}, \pi_{(011)}, \pi_{(111)}\right) \\
&= \frac{\sum_{i=1}^{n} \boldsymbol{a}_i}{\sum_{i=1}^{n} \sum_{j=1}^{r} a_{ij}} \\
&= \frac{\sum_{i=1}^{n} \boldsymbol{a}_i}{\sum_{i=1}^{n} m_i} \\
&= \left(\tfrac{3}{13}, \tfrac{0}{13}, \tfrac{5}{13}, \tfrac{1}{13}, \tfrac{0}{13}, \tfrac{0}{13}, \tfrac{0}{13}, \tfrac{4}{13}\right).
\end{aligned}$$

3 Model specification

In the above hypothetical example, the genotype frequencies were trivial to calculate because the genotype counts and MOIs were known. In reality, however, the genotype counts and MOIs are unobserved and unknown. To infer the genotype frequencies, a model, which sums over and all possible MOIs, is fit to the observed data. Let $\boldsymbol{H}$ be the matrix in which all possible patient level vectors of genotype counts given a specified MOI is stored (see Section 1.2). The posterior probability of the genotype frequencies, $\boldsymbol{\pi} = (\pi_1 \cdots \pi_r)$, conditional on the observed data, $\boldsymbol{Y}$, is thus given by:

$$Pr\left(\boldsymbol{\pi}|\boldsymbol{Y}\right) \propto \prod_{i=1}^{n} \left\{ \sum_{m_i=1}^{m_{max}} \left(\sum_{g=1}^{k} Pr\left(\boldsymbol{y}_i|\boldsymbol{h}_g\right) Pr\left(\boldsymbol{h}_g|m_i, \boldsymbol{\pi}\right) \right) Pr\left(m_i\right) \right\} Pr\left(\boldsymbol{\pi}\right).$$

$Pr\left(\boldsymbol{y}_i|\boldsymbol{h}_g\right)$ is modelled using an identity function:

$$I\left(\boldsymbol{y}_i|\boldsymbol{h}_g\right) = \begin{cases} 1 & \text{if } \boldsymbol{h}_g \text{ is compatible with } \boldsymbol{y}_i, \\ 0 & \text{otherwise.} \end{cases}$$

For example, if $\boldsymbol{y}_i = (0.5, 0, 0)$ and $\boldsymbol{h}_g = (7, 1, 0, 0, 0, 0, 0, 0)$, where $\boldsymbol{h}_g = \left(n_{(000)}, n_{(100)}, n_{(010)}, n_{(001)}, n_{(110)}, n_{(101)}, n_{(011)}, n_{(111)}\right)$ and $n_{(100)}$ is the number of clones with genotype 100 etc., then $I\left(\boldsymbol{y}_i|\boldsymbol{h}_g\right) = 1$.

Each patient level vector of genotype counts, $\boldsymbol{h}_g$, is assumed to be a realisation of size m_i from a multinomial distribution with probability $\boldsymbol{\pi}$. The prior on $\boldsymbol{\pi}$ is Dirichlet, where $\alpha_1 = \alpha_2 = \cdots = \alpha_r = 1$. The prior on m_i is one of four possible distributions: Uniform, where $m_i \in \{1, m_{max}\}$; a truncated Poisson distribution, where $m_i \in \{1, m_{max}\}$, and the parameter of the distribution is set equal to the reported mean MOI; a truncated negative Binomial distribution, where $m_i \in \{1, m_{max}\}$, and the mean parameter of the distribution is set equal to the reported mean MOI and the dispersion factor is $\frac{1}{2}$; or a truncated Geometric distribution, where $m_i \in \{1, m_{max}\}$, and the parameter of the

truncated Geometric distribution is set equal to the reciprocal of the reported mean MOI. The prior that provides the best model fit (see Section 4) is selected. In practice, the normalising constants that result from truncation cancel in the Metropolis-Hastings step (Section 3.1). The truncated term is thus dropped from descriptions in Additional file 2.

3.1 Sampling algorithm

The double summation over the possible patient level vectors of genotype counts and MOI values is computationally expensive. The model approximates $Pr\left(\boldsymbol{\pi}|\boldsymbol{Y}\right)$ by averaging over successive samples of patient genotype count vectors, $\boldsymbol{a_i}$, and MOIs, m_i. The sampling procedure consists of a Metropolis-Hastings step embedded within a Gibbs sampler. Starting with an initial estimate of $\boldsymbol{\pi}^0$, $\boldsymbol{m}^0$ and $\boldsymbol{A}^0$, at $t=0$, it proceeds as follows[6]:

1. Update $\boldsymbol{\pi}^t$ given $\boldsymbol{m}^t$, $\boldsymbol{A}^t$, and $\boldsymbol{Y}$: $\boldsymbol{\pi}^{t+1}$ is drawn directly from its full conjugate conditional distribution:

$$Pr\left(\boldsymbol{\pi}^{t+1}|\boldsymbol{A}^t, \boldsymbol{m}^t, \boldsymbol{Y}\right) \propto \text{Dir}\left(\alpha_1 + \sum_{i=1}^{n} a_{i\,1}^t, \cdots, \alpha_r + \sum_{i=1}^{n} a_{i\,r}^t\right).$$

2. Propose an update, $\boldsymbol{m}^*$, given $\boldsymbol{m}^t$ and $\boldsymbol{Y}$: for $i=(1,\cdots,n)$, if $m_{min} < m_i^t < m_{max}$, where $m_{min}=2$, for observations with a least one mixed SNP, and $m_{min}=1$ otherwise, a proposed MOI, $m_i^* = m_i^t \pm 1$, is selected with probability $\frac{1}{2}$. If $m_i^t = m_{min}$, $m_i^* = m_i^t + 1$; if $m_i^t = m_{max}$, $m_i^* = m_i^t - 1$.

3. Propose an update, $\boldsymbol{A}^*$, given $\boldsymbol{m}^*$, $\boldsymbol{A}^t$, $\boldsymbol{m}^t$, $\boldsymbol{\pi}^{t+1}$, and $\boldsymbol{Y}$: for each individual patient, a new genotype count vector is proposed by either adding or subtracting a clone (depending on m_i^*) from the current genotype count vector.

   If a clone is added, $m_i^* = m_i^t + 1$, the genotype of the new clones is drawn from a multinomial distribution with renormalised probability vector, $\boldsymbol{\pi}_{add}^{t+1}$, such that incompatible genotypes have zero probability:

$$\boldsymbol{a}_i^* = \boldsymbol{a}_i^t + \text{Mn}\left(1, \boldsymbol{\pi}_{add}^{t+1}\right),$$

   where Mn denotes the multinomial distribution. If a clone is subtracted, $m_i^* = m_i^t - 1$, the genotype of the subtracted clone is drawn from a multinomial distribution whose probability vector, $\boldsymbol{\pi}_{sub}^{t+1} = f(\boldsymbol{a}_i^t)$, is renormalised such that the

[6]Imputation of incomplete data: because observed data are conditional on the genotype counts (see Figure A1.1), by specifying an initial estimate $\boldsymbol{A}^0$, missing data are assigned initial estimates. Moreover, as the sampling algorithm proceeds ($t > 0$), each time new set of genotypes ($\boldsymbol{A}^t$) is sampled, new imputed values for the missing data are assigned.

probability of subtracting an existing clone that will render the ensuing genotype count incompatible with the observed data is zero:

$$\boldsymbol{a}_i^* = \boldsymbol{a}_i^t - \text{Mn}\left(1, \boldsymbol{\pi}_{sub}^{t+1}\right).$$

4. Accept or reject proposals, $\boldsymbol{A}^*$ and $\boldsymbol{m}^*$: the proposed MOI estimates and genotype counts are rejected or accepted based on a Metropolis-Hastings step, which is repeated for $i = 1, \cdots, n$:

$$Pr\left(\text{Accept } \boldsymbol{a}_i^*, m_i^*\right) = \min\left\{1, \frac{Pr\left(\boldsymbol{a}_i^*, m_i^* | \boldsymbol{y}_i, \boldsymbol{\pi}^{t+1}\right) Q\left(m_i^t | m_i^*, \boldsymbol{y}_i\right) Q\left(\boldsymbol{a}_i^t | m_i^t, \boldsymbol{a}_i^*, m_i^*, \boldsymbol{y}_i, \boldsymbol{\pi}^{t+1}\right)}{Pr\left(\boldsymbol{a}_i^t, m_i^t | \boldsymbol{y}_i, \boldsymbol{\pi}^{t+1}\right) Q\left(m_i^* | m_i^t, \boldsymbol{y}_i,\right) Q\left(\boldsymbol{a}_i^* |, m_i^*, \boldsymbol{a}_i^t, m_i^t, \boldsymbol{y}_i, \boldsymbol{\pi}^{t+1}\right)}\right\}.$$

where $Q(\cdot)$ denotes proposal density[7].

The algorithm is run sufficiently for it to converge and the burn in is discarded. The sample $\{\boldsymbol{\pi}\}$ is obtained by discarding $\boldsymbol{A}$ and $\boldsymbol{m}$ from the joint sample $\{\boldsymbol{\pi}, \boldsymbol{A}, \boldsymbol{m}\}$. The samples $\{\pi_1, \cdots, \pi_r\}$, which approximate the marginal distributions of the full genotype frequency posterior distribution, are obtained from $\{\boldsymbol{\pi}\}$.

4 Model checking

Several approaches, recommended by Gelman et al. [Gelman A, Carlin JB, Stern HS, Rubin DB: Bayesian Data Analysis. 2nd edition. CRC Press; 2004], are used to check the model (see Additional file 2). For completeness, mathematical details of any modifications of the methods reported by Gelman et al. are provided below.

4.1 Model error

To summarise model error, Gelman et al. recommend using the estimated average deviance:

$$\hat{D}_{\text{avg}}(\boldsymbol{Y}) = \frac{1}{L}\sum_{l=1}^{l} D(\boldsymbol{Y}, \boldsymbol{\pi}^l),$$

where $D(\boldsymbol{Y}, \boldsymbol{\pi}^l) = -2 \log Pr(\boldsymbol{Y}|\boldsymbol{\pi}^l)$, and L is the total number of posterior samples. Since $D(\boldsymbol{Y}, \boldsymbol{\pi}^l)$ requires summing over all possible genotype counts ($\boldsymbol{A}$) and MOIs ($\boldsymbol{m}$), deviance is based on $\boldsymbol{A}^l, \boldsymbol{\pi}^l$ and $\boldsymbol{m}^l$:

$$\begin{aligned} D(\boldsymbol{A}^l, \boldsymbol{\pi}^l, \boldsymbol{m}^l) &= -2 \log Pr(\boldsymbol{Y}, \boldsymbol{A}^l | \boldsymbol{\pi}^l, \boldsymbol{m}^l) \\ &= -2 \log \left[Pr(\boldsymbol{Y}|\boldsymbol{A}^l) Pr(\boldsymbol{A}^l | \boldsymbol{\pi}^l, \boldsymbol{m}^l)\right] \\ &= -2 \log Pr(\boldsymbol{A}^l | \boldsymbol{\pi}^l, \boldsymbol{m}^l), \text{since all } \boldsymbol{a}_i^l \text{ are compatible with } \boldsymbol{y}_i, \end{aligned}$$

[7]The proposal densities are discussed in Steps 2 and 3.

4.2 Posterior predictive check

Replicate datasets, $\boldsymbol{Y}^{rep}$, are generated from the posterior predictive sample of patient MOIs and genotype frequencies, $\{\boldsymbol{\pi}^l, \boldsymbol{m}^l\}$, where $l = 1, \cdots, L$, and L is the total number of posterior samples. A replicate dataset, $\boldsymbol{Y}^{rep\,l}$, is generated by drawing replicate genotype count, $\boldsymbol{a}_i^{rep\,l}$, one patient at a time, from a multinomial distribution:

$$\boldsymbol{y}_i^{rep\,l} = f(\boldsymbol{a}_i^{rep\,l}), \text{ where } \boldsymbol{a}_i^{rep\,l} \sim \text{Mn}\left(m_i^l, \boldsymbol{\pi}^l\right).$$
