# Supplementary material for: Estimation of malaria haplotype and genotype frequencies: a statistical approach to overcome the challenge associated with multiclonal infections
Source: Malar J. 2014 Mar 17;13:102. doi: 10.1186/1475-2875-13-102 (PMC4004158; doi:10.1186/1475-2875-13-102)
Supplement: Additional file 2 — Full details of the simulation studies and analyses of the field data. [file 1475-2875-13-102-S2.pdf]

Additional file 2

1 Generation of simulated data

1.1 Generation of simulated data for model validation and sensitivity analysis

Unless otherwise stated, 100 blood samples were simulated per dataset. A vector of 100 multiplicities of infection (MOIs) were generated by sampling from a Poisson distribution with parameter equal to three. Draws of zero, which represent uninfected individuals, were discarded and the MOI resampled. For each infected blood sample, the genotypes of the clones were drawn from a multinomial distribution (the number of clones and, therefore, size of the draw from the multinomial distribution were equal to the MOI). The probability of drawing a given genotype was set equal to a vector of genotype frequencies[1], which was sampled from a uniform Dirichlet distribution. Having assigned genotypes to each individual clone within each infected blood sample, the empirical genotype frequencies (the proportion of each genotype in the dataset) were calculated. For each blood sample, an observation was generated assuming 100% detectability. In other words, minority alleles were always detected, regardless of how low their frequency.

1.2 Generation of simulated data to provide insight into the accuracy of the analyses of the field data

To ensure the simulated data resembled the field data, they were generated as above (Section 1.1) with the following modifications. For each of the five sites, the number of blood samples simulated was equal to the number of blood samples in the field data. Missing data were introduced into the simulated data at locations that correspond with missing data in the field data. For each site, the vector of MOIs were generated by sampling from a Geometric distribution[2] with parameter equal to the reciprocal of the

[1] A genotype frequency is an estimate of the proportion of parasite clones in the parasite population that carry a given genotype.

[2] In the case of data simulated to resemble field data, MOIs were sampled from a Geometric distribution because the Geometric distribution was found to provide the best fit to the field data (see Section 3).

reported mean MOI at that site. The probability of drawing a given genotype was set equal to the frequency point estimates derived from the analysis of the field data given the reported mean MOI at each site.

2 Model validation and sensitivity analysis

It is important to note that the results in the following Section were averaged over the genotype frequency estimates[3] within each analysis, as well as across the analyses of ten different datasets for each combination of variables investigated. Doing so accounts for variation in genotype frequencies and datasets. However, it may also mask genotype specific effects. To see how average results translate into specific estimates and datasets, for each experiment, plots of empirical genotype frequencies, their estimates and their 95% credible intervals[4] were generated and checked for every single genotype given one to five single nucleotide polymorphisms (SNPs) (62 genotypes in total). Examples plots are included. Throughout this Additional file, precision is defined as the standard deviation of the MCMC genotype frequency sample (low values correspond to precise estimates). Accuracy is defined as the absolute error between the frequency estimate and the true, empirical frequency, which was known since the data were simulated (again, low values correspond to accurate estimates)[5]. Unless stated otherwise, all chains were run for 50,000 iterations (see Section 2.1).

2.1 Assessing model convergence

To monitor model convergence, qualitative visual checks of the MCMC trace and log likelihood plots were firstly used. Second, the within and between sequence variances of three parallel chains initialised from sparse initial genotype frequency values were compared, in order to estimate the potential scale reduction for each individual genotype frequency, using the method recommended by Gelman et al. (Gelman A, Carlin JB, Stern HS, Rubin DB: Bayesian Data Analysis. 2nd edition. CRC Press; 2004). The potential scale reduction is an indicator of the factor by which the discrepancy in variation might be reduced if the current chain were continued for an infinite number of iterations. A value close to one supports the conjecture that the chain has converged. Gelman et al. advise running the chain long enough to reduce all scale reduction estimates to 1.1, with higher

[3]Frequency point estimates were defined by the medians of the Markov chain Monte Carlo (MCMC) frequency samples.

[4]A credible interval is the Bayesian equivalent of a confidence interval. The credible intervals reported throughout this study extended from the 0.025 percentile of each MCMC frequency sample to the 0.975 percentile.

[5]Accuracy and precision tend to be smaller for four or more SNPs because the frequency mass is shared over a greater number of genotypes (the number of genotypes increases exponentially with the number of SNPs genotyped).

precision for final analyses. For one to five SNPs, ten datasets were analysed[6]. A scale reduction is estimated for each individual genotype within each dataset. The maximum scale reduction is reported. The overdispersed frequency vectors were generated by setting all but one of the initial frequencies (selected at random) to 0.02. The remaining frequency was fixed such that the total frequencies summed to one. When only one SNP was genotyped, one of the chains was initialised from equal initial frequencies (both equal to $\frac{1}{2}$). 50,000 iterations were found to be sufficient based on a potential scale reduction < 1.05 (see Table A2.1).

	No. of MCMC iterations		
No. of SNPs	10,000	20,000	50,000
1	1.002	1.001	1.001
2	1.015	1.023	1.007
3	1.081	1.069	1.023
4	1.221	1.074	1.035
5	1.263	1.084	1.045

Table A2. 1: Assessing convergence of the MCMC algorithm: the maximum estimated scale reduction as a function of the number of SNPs and the number of MCMC iterations. A value close to one supports the conjecture that the chain has converged.

2.2 Comparing point estimates

The impact of initialising the MCMC algorithm at different initial genotype frequency estimates on the final frequency estimates was investigated. For a single SNP, datasets were analysed by three parallel chains: two had overdispersed initial frequencies (generated by setting all but one of the frequencies to 0.02); the other had equal frequencies (both equal to $\frac{1}{2}$). In the case of two or more SNPs, five chains were compared. The initial genotype frequency vectors for each of the five chains were selected at random, without replacement, from a set of overdispersed initial frequency vectors (again, generated by setting all but one of the frequencies to 0.02) plus a vector of equal frequencies. To evaluate the impact of intialising the MCMC at different initial frequency vectors, in addition to individual genotype plots (for example, Figure A2.1), for each dataset, the difference between estimates obtained from different chains was calculated. The final frequency estimates were robust to changes in the initial frequency estimates. The difference between estimates obtained from different chains was small (mean difference 4.75×10^{-3}), ranging from 3.76×10^{-5}, to 1.84×10^{-2} for genotype 00000 (dataset B, Figure A2.1).

[6]Throughout the entire simulation study, results were averaged over ten datasets for each unique combination of variables investigated. However, in the interest of brevity, this is only referred to explicitly hitherto.

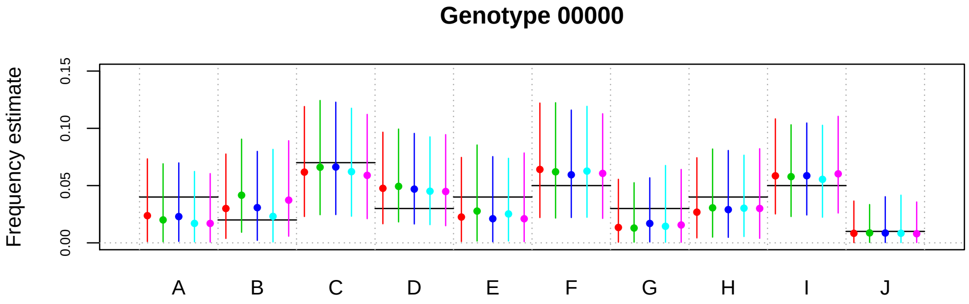

Figure A2.1: The final frequency estimates (points) and their 95% credible intervals (vertical lines) for genotype 00000, obtained from five separate analyses (five chains, highlighted in different colours, each initiated at a different vector of overdispersed initial frequency estimates) for ten different datasets (A-J). The initial frequency vectors were generated by fixing all but one (selected at random) of the genotypes to 0.02. The randomly selected genotype was set to ensure each vector summed to one. For each dataset, the true empirical frequency is depicted by a black horizontal bar.

2.3 Assessing computational speed, accuracy and precision as a function of the simulated data.

To test the speed, precision and accuracy of the model as a function of data and SNPs, simulated datasets with 50, 100 and 1000 blood samples, and one to five SNPs were analysed using the model. The datasets were subsequently analysed using a counting method (all discernibly multiclonal blood samples were discarded and proportions calculated given the remaining sub-sample of the data). Any genotype frequencies that were not calculable using the counting method were set to zero. Doing so ensured that accuracy was averaged over the same number of genotypes in each case.

It terms of computational speed, the algorithm scaled efficiently with the number of SNPs, since the summation over all possible MOIs and genotype combinations was approximated (Table A2.2). The precision and accuracy of the statistical estimates increased with the number of patient blood samples. This demonstrated the validity of the model, in that the estimates converged on the truth as the evidence increased. Importantly, the accuracy of the statistical estimates was superior to that obtained using the counting method.

2.4 Effect of suboptimal detectability of clones

To investigate the impact of suboptimal detectability of alleles on frequency estimates, for one to five SNPs, ten cohorts of infected blood samples were simulated assuming an average MOI of one, three, five and seven. Having simulated each cohort, datasets were

No. SNPs	No. samples	Statistical method CPU time (s)	Precision	Accuracy	Counting method Accuracy
	50	56	0.034	0.015	0.075
1	100	74	0.027	0.016	0.101
	1000	310	0.010	0.006	0.083
	50	82	0.038	0.022	0.092
2	100	120	0.034	0.022	0.058
	1000	580	0.011	0.007	0.046
	50	120	0.039	0.035	0.074
3	100	180	0.029	0.022	0.047
	1000	1100	0.010	0.007	0.026
	50	190	0.032	0.024	0.053
4	100	290	0.024	0.017	0.040
	1000	2000	0.008	0.007	0.014
	50	290	0.021	0.017	0.040
5	100	480	0.017	0.013	0.028
	1000	3700	0.007	0.006	0.010

Table A2. 2: Model performance as a function of the simulated data. CPU time (s): central processing unit time in seconds. Low values indicate speed, accuracy and precision (for definitions of accuracy and precision, see the introductory paragraph to Section 2).

generated from the infected blood samples, firstly given 100% detectability; secondly, given 90% detectability (minority alleles that contributed less than 10% to a given SNP evaded detection); and finally, given 70% detectability (minority alleles that contributed less than 30% to a given SNP evaded detection). All the datasets (600 in total) were analysed assuming 100% detectability.

The results were less accurate given 70% detectability; for example, see Figure A2.2: all but two of the fifteen 95% credible intervals, out of a total of 120, that do not contain the empirical frequency correspond with datasets generated assuming 70% detectability. The difference in mean accuracy between 100-90% detectability, however, was small (Figure A2.3). Furthermore, given three or more SNPs, and an average MOI equal three or one, there is little difference between 100-70% detectability. Unsurprisingly, the detrimental effect of suboptimal detectability is more profound when the average MOI increased, since a higher proportion of infected blood samples were affected. Relative to accuracy, suboptimal detectability appears to have a small positive effect on precision, which is clearly spurious, and is seemingly due to the relative decline in the number of mixed SNPs[7] (Figure A2.3).

Suboptimal detectability primarily affected datasets comprised of three or fewer SNPs (Figure A2.3). In addition to the method used to define accuracy (see introductory para-

[7]Mixed SNPs were single nucleotide polymorphisms (SNPs) at which both a sensitive and resistance markers were simultaneously detected.

graph to Section 2), this may, in part, be due to the method used to generate the data. The data were generated using population genotype frequencies drawn from a uniform Dirichlet distribution. Since the number of possible genotypes increases exponentially with the number of SNPs genotyped, genotypes with frequencies close to 1 tend to be rare in datasets comprised of four or more SNPs. Since the likelihood that a single SNP is dominated by a single allele (detectability applies to alleles, not genotypes) is lower in a dataset comprising a large number of genotypes over which mass is evenly distributed, suboptimal detectability primarily affected datasets comprised of three or fewer SNPs, especially when mass is unevenly distributed (for example, see the estimate for genotype 010, cohort 26, average MOI 5, Figure A2.2).

2.5 Sensitivity of the model to the assumed distribution over the multiplicity of infection (MOI)

For one to five SNPs, ten datasets, each with multiplicities of infection (MOIs) generated from a Poisson distribution with parameter equal to three, were analysed multiple times. The first analysis was performed under the assumption that the distribution over the MOI was Poisson. Three subsequent analyses were performed assuming the distribution over the MOI was Uniform over one to eight, negative Binomial with mean parameter equal to three, and the dispersion factor equal to a half; and Geometric with parameter equal to a third. Unsurprisingly given the data generating process, on average, the model with a Poisson distribution over the MOI gave rise to the most accurate genotype frequencies (Table A2.3). The differences between estimates generated under the Uniform, Poisson and negative Binomial distributions were relatively small (for example, see Figure A2.4). Relative to accuracy, the precision of genotype frequency estimates appeared to be robust to misspecification of the prior distribution over the MOI (Table A2.4). The mean estimated average deviance was used to compare models with different MOI priors: Uniform, Geometric, Poisson and negative Binomial. According to this method, aside from datasets with five markers, the Geometric prior (the model with the lowest average deviance), provides the best fit to the data (Table A2.5). This is surprising; the Poisson prior is expected to give the best fit, since the data were generated using a Poisson distribution. The unexpected result may reflect the fact that the estimated average deviance was based on the likelihood of the estimated genotype counts, rather than the simulated data themselves (see Additional file 1). The sensitivity of simulated data analysis to misspecification of the prior distribution, and the unexpected result based on deviance, motivates additional model checks to ascertain the prior distribution that provides the best fit to the field data (see Section 3).

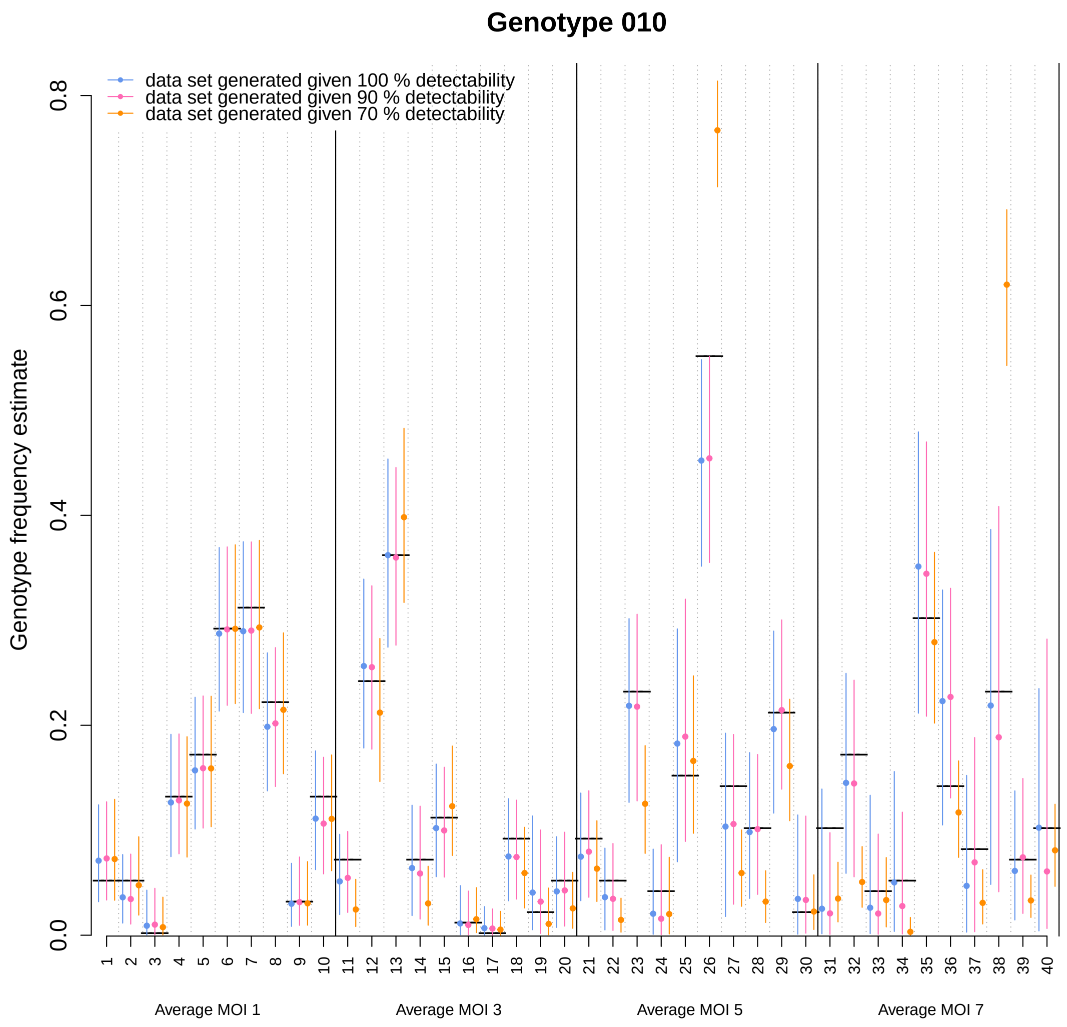

Figure A2.2: Genotype frequency estimates (points) and their 95% credible intervals (vertical lines) given suboptimal genotyping sensitivity. Ten cohorts of one hundred infected blood samples were generated per specified MOI (40 cohorts in total: 1-10, average MOI of one; 11-20, average MOI of three; 21-30, average MOI of five; 31-40 average MOI of seven). To enable comparison between results given different limits of detectability, three datasets were generated per cohort: one given 100% detectability (blue), another given 90% detectability (pink), and another given 70% detectability (orange). Each of the three datasets generated from a common cohort have the same empirical 010 genotype frequency (black horizontal bar), since the genotypes in the infected blood samples remained the same despite changing the detectability. The datasets were all analaysed assuming 100% detectability.

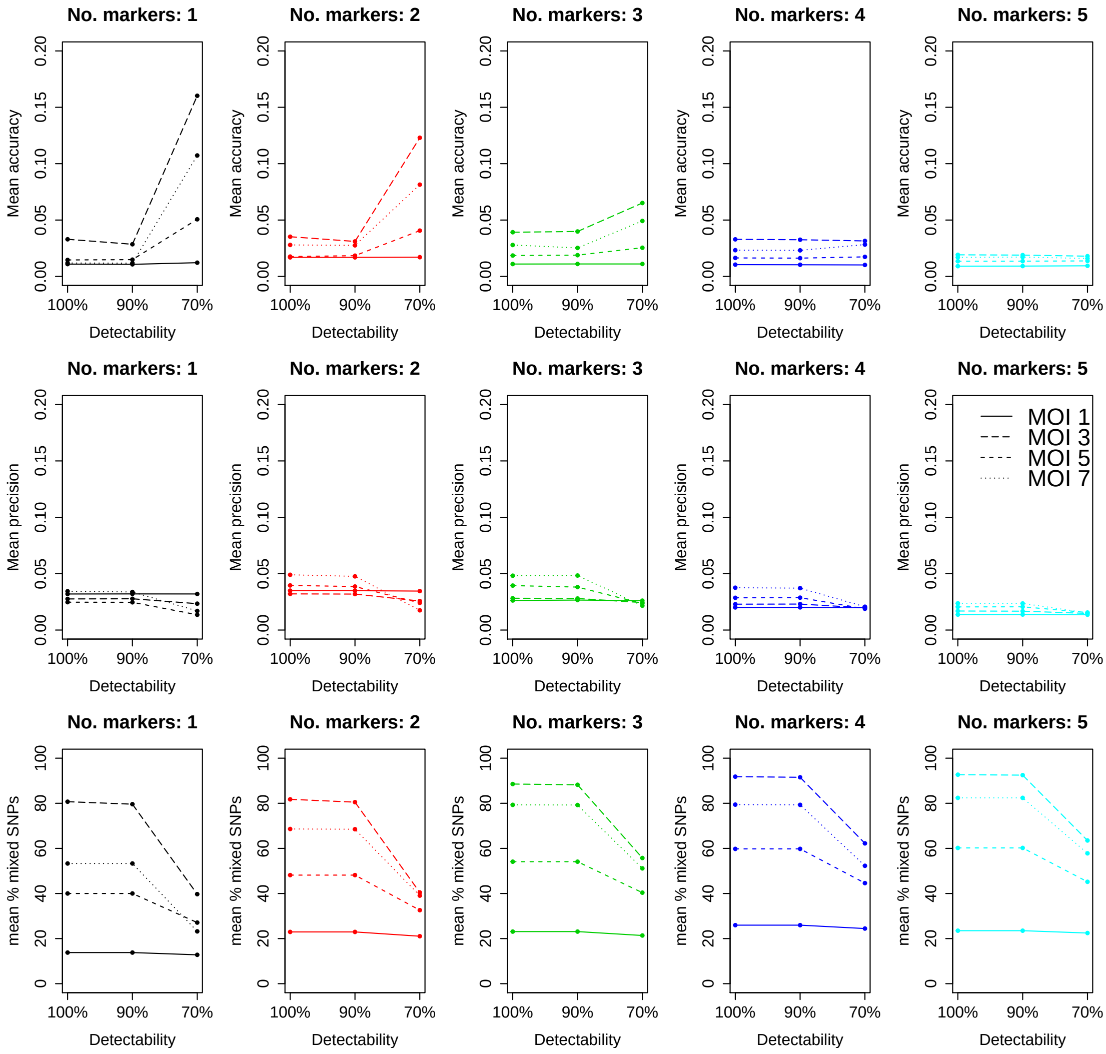

Figure A2. 3: The impact of suboptimal detectability on the accuracy and precision of the frequency estimates, and the mean percentage of mixed SNPs per dataset. Low values for accuracy and precision indicate high accuracy and precision (see see the introductory paragraph to Section 2). For one to five SNPs and specified MOI, ten cohorts of one hundred infected blood samples were generated. Three datasets were generated per cohort: one given detectability equal to 100%, 90% and 70%. The datasets (600 in total) were analysed assuming 100% detectability.

2.6 Sensitivity of the model to the MOI prior distribution parameter

For one to five SNPs, ten datasets, each with MOIs generated from a Poisson distribution with parameter equal to three, were analysed given a Poisson parameter[8] equal

[8] The Poisson parameter was referred to as the mean MOI in the main manuscript since it takes the value of the mean MOI specified by the investigator.

	MOI prior distribution			
No. of SNPs	Uniform	Poisson	nBinomial	Geometric
1	0.051	**0.016**	**0.016**	0.024
2	0.032	**0.023**	0.027	0.028
3	0.027	**0.022**	0.023	0.023
4	**0.017**	0.018	**0.017**	0.018
5	**0.013**	**0.013**	**0.013**	**0.013**

Table A2.3: The impact of MOI prior misspecification on the mean accuracy of the frequency estimates. Low values (highlighted in bold type) indicate high accuracy.

	MOI prior distribution			
No. of SNPs	Uniform	Poisson	nBinomial	Geometric
1	**0.024**	0.027	0.030	0.031
2	0.035	**0.034**	0.036	0.037
3	**0.028**	0.029	0.030	0.030
4	**0.024**	**0.024**	**0.024**	**0.024**
5	**0.017**	**0.017**	**0.017**	**0.017**

Table A2.4: The impact of MOI prior misspecification on the mean precision of the frequency estimates. Low values (highlighted in bold type) indicate high precision.

	MOI prior distribution			
No. of SNPs	Uniform	Poisson	nBinomial	Geometric
1	184	173	166	**160**
2	510	453	462	**440**
3	840	745	769	**736**
4	1267	1129	1172	**1127**
5	1686	**1503**	1568	1508

Table A2.5: The impact of MOI prior misspecification on the mean estimated average deviance. Low values (highlighted in bold type) indicate good model fit (see Additional file 1 for details about the estimated average deviance).

to one (underestimate), three, and five (overestimate). Unsurprisingly, on average, the correct MOI parameter specification gave rise to the most accurate frequency estimates (Table A2.6). The detrimental effect of overestimating appears to be less than that of underestimating the MOI. In fact, it appeared that no MOI specification was preferable to misspecification (Table A2.6). Contrary to accuracy, MOI overestimation had a favorable effect on the precision of the genotype frequency estimates (Table A2.7). The

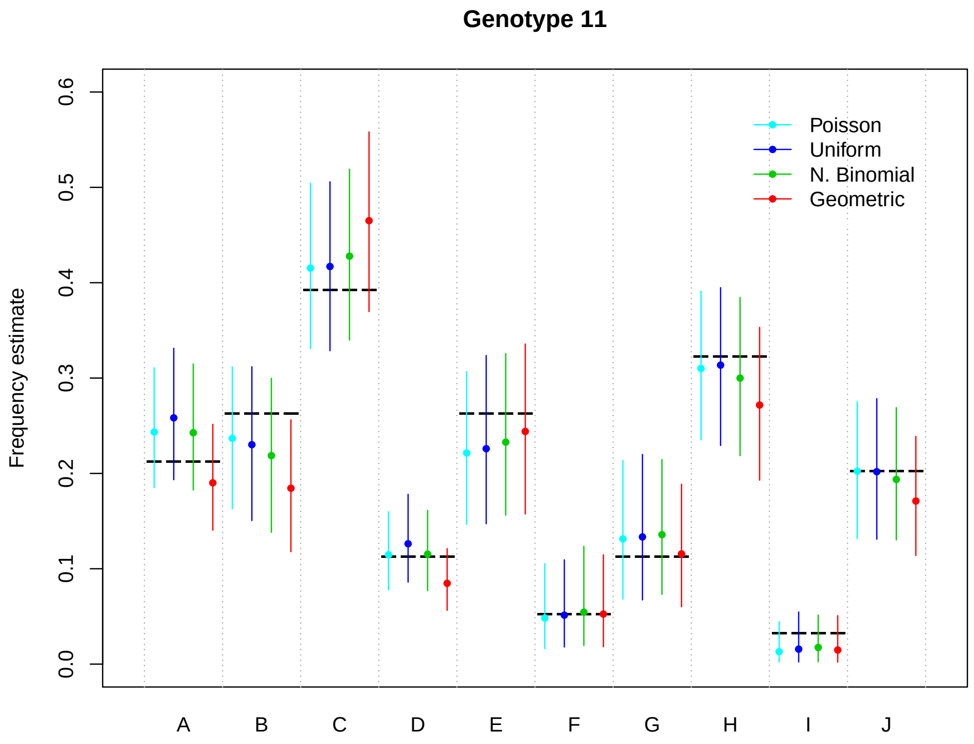

Figure A2. 4: The impact of MOI prior misspecification on frequency estimates (points) and their 95% credible intervals (vertical lines) for the genotype 11, colour coded by the MOI prior distribution, across ten different datasets (A-J). The empirical frequency is denoted by the black horizontal bar.

reason for this is simple: MOI overestimation augments the number of clones per blood sample, thus leading to a greater number of genotype assignments on which to base the genotype frequencies. Sensitivity of the model to the Poisson parameter motivates the repeat analysis of field data, each time varying the MOI prior parameter, in order to establish the sensitivity of the results (for example, see Section 3). Based on the accuracy when the MOI is unspecified compared with misspecified, if the mean MOI is unknown, a uniform prior is recommended.

2.7 Sensitivity of the model to imputing missing data

To investigate the impact on estimated genotype frequencies of missing data, incomplete datasets were analysed. For one to five SNPs, ten datasets were each analysed multiple times. In the first instance, the datasets were analysed intact (no missing data). Thereafter, data were erased from 25%, 50% and 75% of the blood samples selected at random (Figure A2.5). The number of missing data per blood sample and the SNPs erased were

No. of SNPs	MOI unspecified (Uniform prior)	MOI (Poisson prior parameter) Correct	Underestimate	Overestimate
1	0.051	**0.016**	0.060	0.058
2	0.032	**0.023**	0.044	0.032
3	0.027	**0.023**	0.028	0.026
4	**0.017**	**0.017**	0.021	**0.017**
5	**0.013**	**0.013**	0.014	**0.013**

Table A2.6: The impact of MOI misspecification on the mean accuracy of the frequency estimates. Low values indicate high accuracy. For comparison, the mean accuracy obtained from the analysis assuming a Uniform prior, for which no MOI was specified, is included.

No. of SNPs	MOI unspecified (Uniform prior)	MOI (Poisson prior parameter) Correct	Underestimate	Overestimate
1	0.024	0.027	0.034	**0.021**
2	0.035	0.035	0.036	**0.032**
3	0.028	0.029	0.029	**0.027**
4	0.024	0.024	0.025	**0.023**
5	0.017	0.017	**0.016**	**0.016**

Table A2.7: The impact of MOI misspecification on the mean precision of the frequency estimates. Low values indicate high precision. For comparison, the mean precision obtained from the analysis assuming a Uniform prior, for which no MOI was specified, is included.

also selected at random. Given each level of erosion, the datasets were analysed twice: first, missing data were imputed (see Additional file 1 for mathematical details); second, incomplete blood samples were discarded. Unsurprisingly, the impact of data erosion on the mean accuracy and precision of the genotype frequency estimates was unfavourable (Tables A2.8 and Table A2.9). However, with the exception of the analyses of datasets with a single SNP, in the majority of cases, imputation of incomplete data improved accuracy and precision. To summarise, imputation enabled use of all available data, whereas partial data were squandered when blood samples with incomplete data were discarded; in the case of a single SNP, there was no partial data.

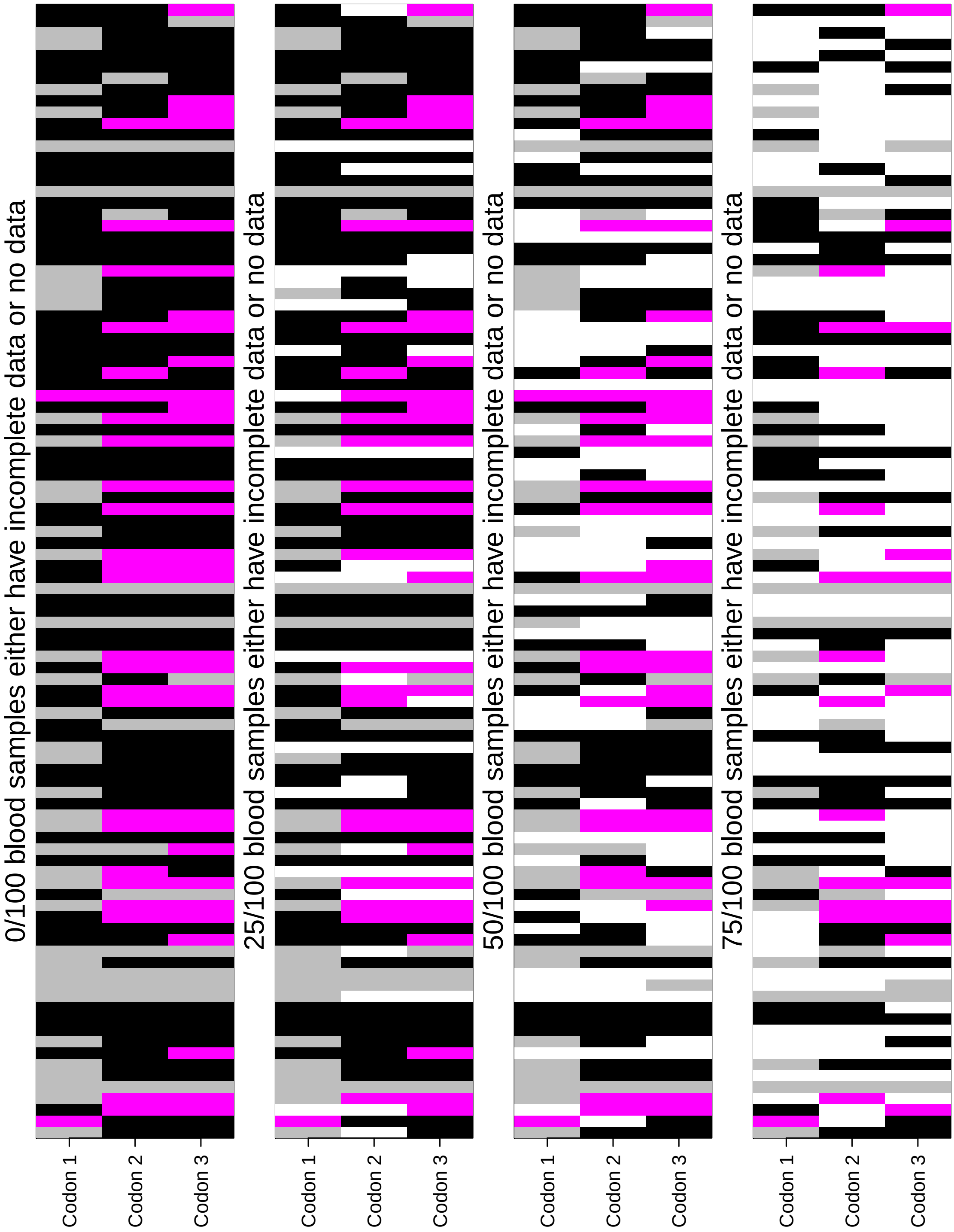

Figure A2.5: An example of data erosion. To investigate the impact on estimated genotype frequencies of missing data, data were erased from 0%, 25%, 50% and 75% of the blood samples, selected at random. Colour code: white, erased datum; pink, sensitive marker; black, resistant marker; grey, both sensitive and resistant markers detected (mixed SNP).

No. of SNPs	% Incomplete blood samples 0	25	50	75
1	16	20 (20)	34 (33)	42 (43)
2	23	28 (28)	32 (33)	40 (42)
3	23	26 (26)	24 (28)	37 (46)
4	17	20 (20)	22 (25)	31 (30)
5	13	14 (15)	15 (16)	17 (19)

Table A2.8: The impact of analysing blood samples with incomplete data on the mean accuracy $\times 10^3$ of the frequency estimates. Low values indicate high accuracy. For comparison, the mean accuracy obtained from the analyses post disposal of blood samples with incomplete data are included in parentheses.

No. of SNPs	% Incomplete blood samples 0	25	50	75
1	27	31 (31)	38 (38)	54 (54)
2	35	39 (40)	42 (45)	54 (64)
3	29	32 (33)	36 (39)	45 (53)
4	24	26 (27)	29 (31)	33 (38)
5	17	18 (18)	19 (20)	21 (23)

Table A2.9: The impact of analysing blood samples with incomplete data on the mean precision $\times 10^3$ of the frequency estimates. Low values indicate high precision. For comparison, the mean precision obtained from the analyses post disposal of blood samples with incomplete data are included in parentheses.

3 Analyses of the field data

3.1 Estimated average deviance

To determine the best model fit to the Ugandan data, a thorough model check was conducted. Estimated average deviance (for a definition, see Additional file 1) was used to compare models with different priors (Uniform, Geometric, Poisson and negative Binomial) over the MOI. According to this method, the model with the lowest average deviance, provides the best fit to the data. The Geometric prior appeared to provide the best fit (Table A2.10). However, given the unexpected result reported in Section 2.5, additional checks based on replicate data were performed (Section 3.3).

	MOI prior distribution			
	Uniform	Poisson	Geometric	nBinomial
Apac	2229	2193	**2146**	2155
Tororo	1802	1808	**1733**	1736
Kanungu	1992	1910	**1786**	1818
Jinja	1649	1565	**1494**	1554
Mubende	2314	2215	**2093**	2138

Table A2.10: Model error, averaged over the posterior distribution. Low values (highlighted in bold) indicate low error and good model fit.

3.2 Replicate data

If a model provides an adequate description of the data generating process, data generated under the model should resemble the field data. To test this premise, the Ugandan datasets were compared with replicate datasets generated from the posterior predictive sample of MOIs and genotype frequencies. Since the posterior samples were large (6000 genotype frequencies and MOIs per MCMC sample), thousands of replicate datasets were compared with a each field dataset. Comparative plots of field and replicate data (for example, see Figure A2.6), and three simple test statistics (summarised as p-values) were used to assess the similarity between field and replicate data. The first test statistic was equal to the fraction of replicate datasets that had a higher percentage of discernibly multiclonal blood samples[9] (Figures A2.7); the second test statistic was equal to the fraction of replicate datasets that had a higher percentage of blood samples with a least one purely resistant SNP (Figures A2.8); and the third test statistic was equal to the fraction of replicate datasets that had a higher percentage of blood samples with a least one purely sensitive SNP (Figures A2.9). A test statistic close to zero or one suggests that, if the model is true, the probability of observing a pattern that resembles the field data in the replicate data is low. A p-value that is neither close to zero or one is, therefore, indicative of model fit with respect to the attribute of the data under consideration. Based on these results, the Geometric prior appears to provide the best fit to the data. However, the predictive applicability of the model with respect to the percentage of discernibly multiclonal blood samples appears to be limited. Since the primary purpose of the model is to estimate genotype frequencies, failure of the model in this regard is not of practical importance. However, use of the model to predict the proportion of discernibly multiclonal blood samples in a dataset is not recommended.

[9]Discernibly multiclonal blood samples were blood samples in which one or more mixed SNPs were detected.

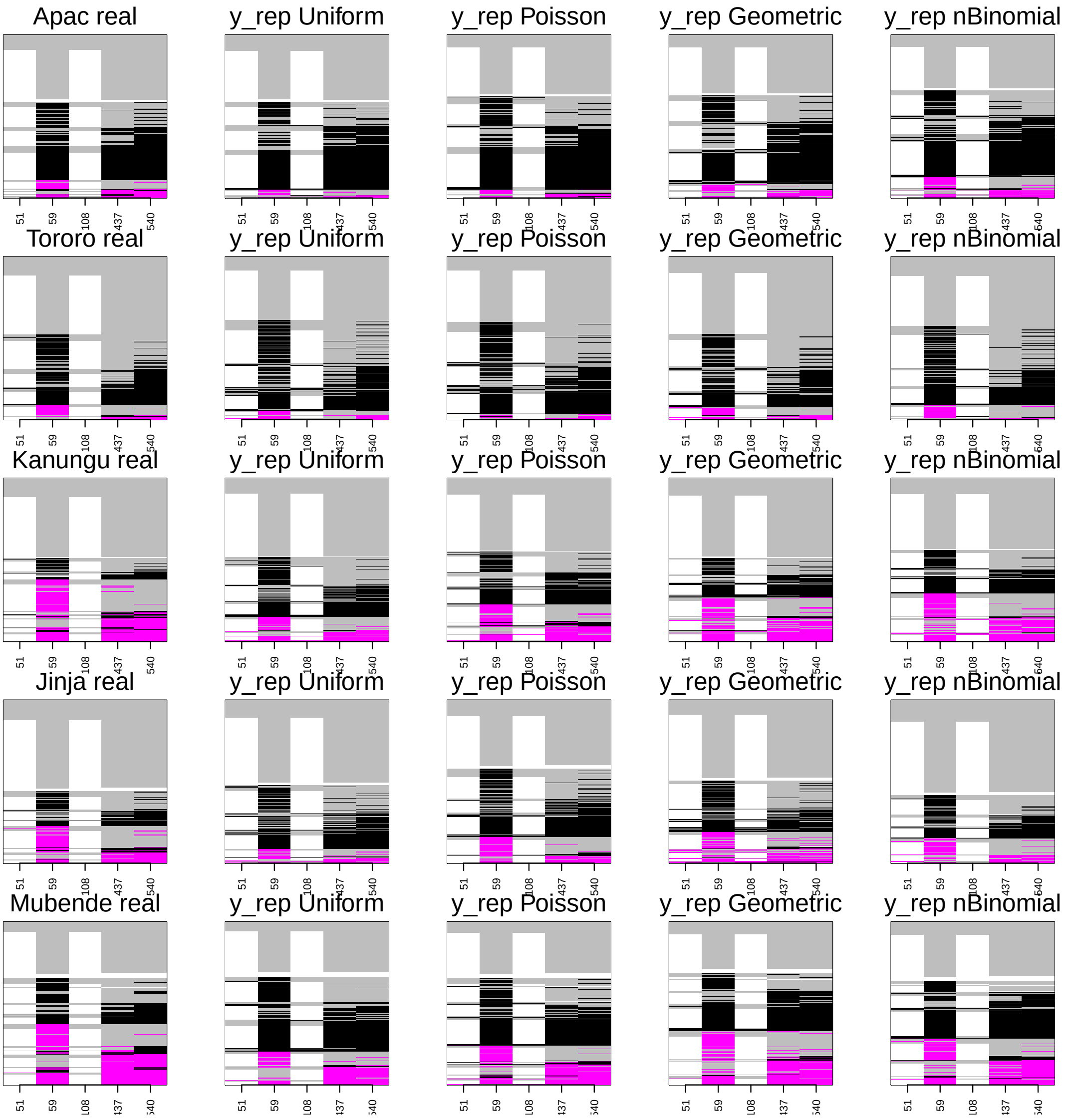

Figure A2.6: Comparitive plots of field and replicate data. Colour code: white, missing datum; pink, sensitive marker; black, resistant marker; grey, both sensitive and resistant markers detected (mixed SNP). The data are arranged into blocks in order to highlight patterns.

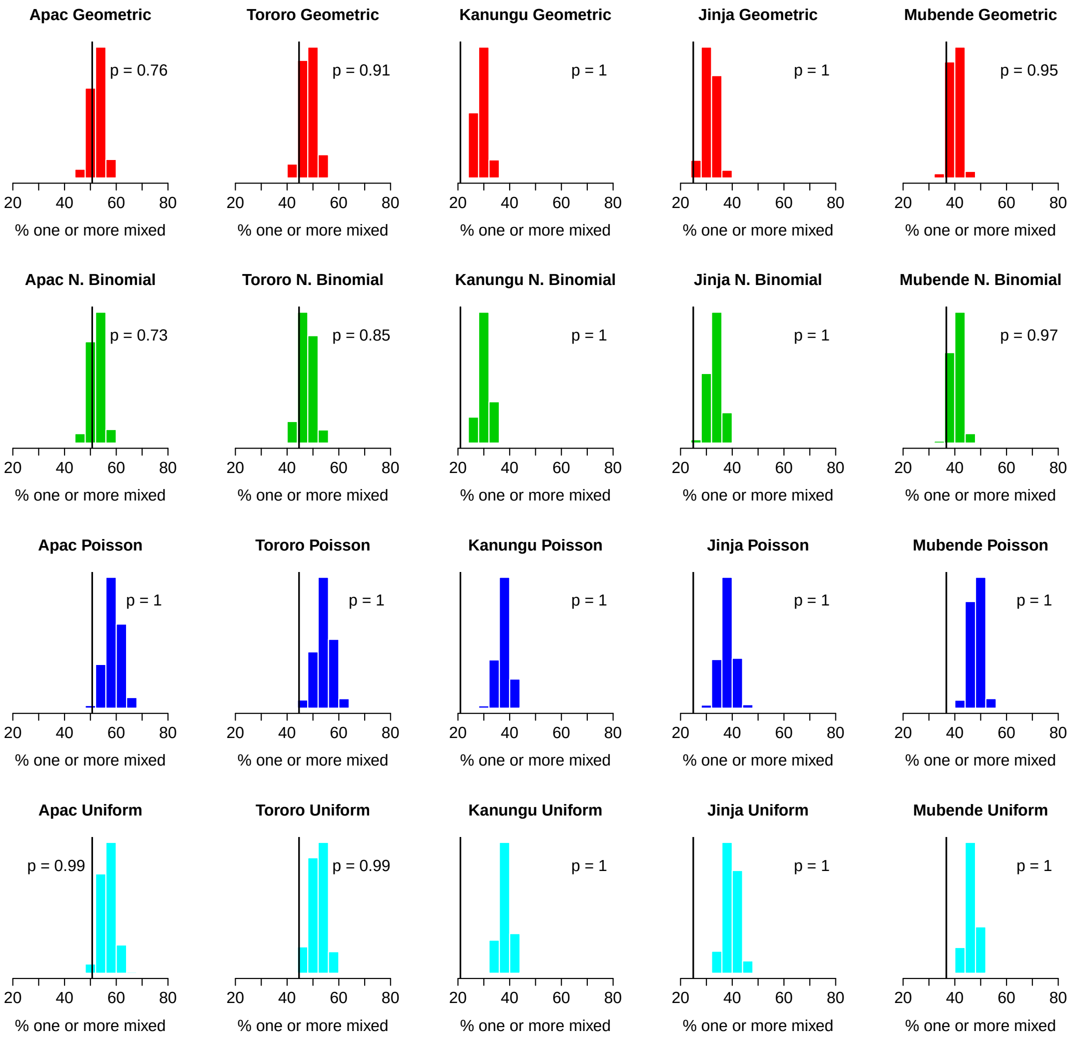

Figure A2.7: The impact of the MOI prior distribution on the posterior predictive distribution of the percentage of blood samples with one or more mixed SNPs in each of the 6000 replicate datasets (datasets are summarised by histograms, which are colour coded by the MOI prior distribution). N. Binomial stands for negative Binomial. The percentage of blood samples with one or more mixed SNPs in the real data is indicated by the black vertical line. The p-value is equal to the fraction (expressed as a decimal number) of replicate datasets that had a higher percentage of blood samples with one or more mixed SNPs than the real data. A p-value that is neither close to zero or one is indicative of model fit with respect to the percentage of blood samples with one or more mixed SNPs.

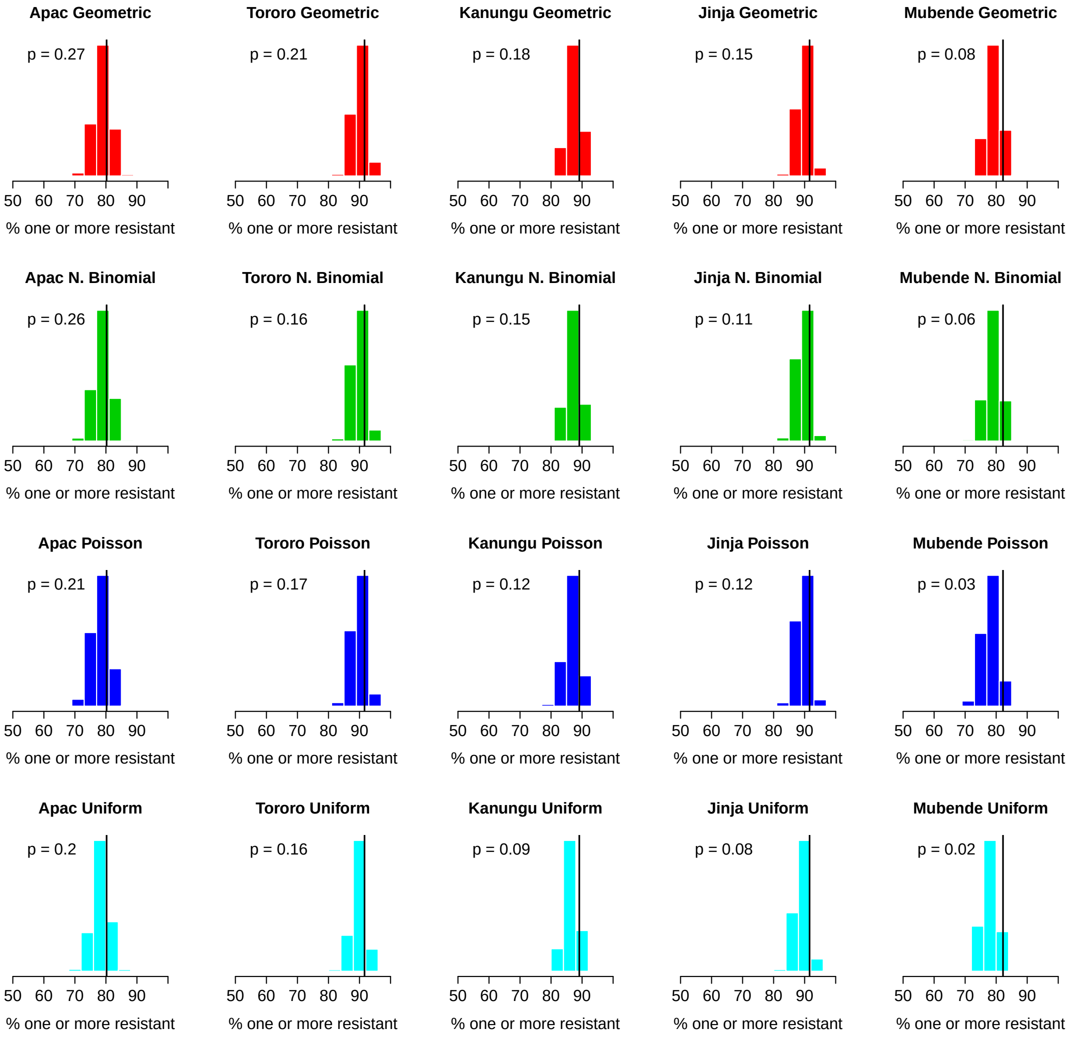

Figure A2.8: The impact of the MOI prior distribution on the posterior predictive distribution of the percentage of blood samples with one or more purely resistant SNPs in each the 6000 replicate datasets (datasets are summarised by histograms, which are colour coded by the MOI prior distribution). N. Binomial stands for negative Binomial. The percentage of blood samples with one or more purely resistant SNPs in the real data is indicated by the black vertical line. The p-value is equal to the fraction (expressed as a decimal number) of replicate datasets that had a higher percentage of blood samples with one or more purely resistant SNPs than the real data. A p-value that is neither close to zero or one is indicative of model fit with respect to the percentage of blood samples with one or more purely resistant SNPs.

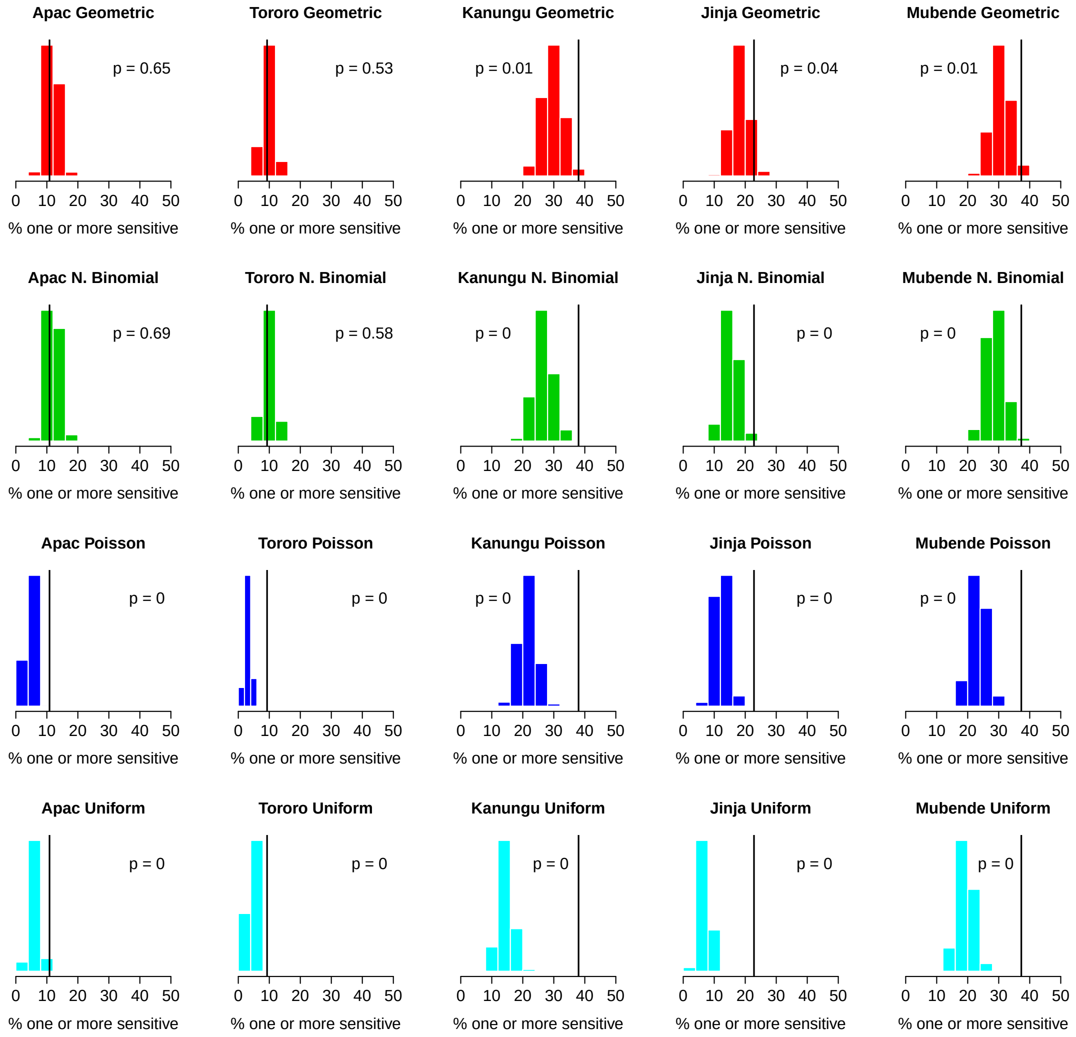

Figure A2.9: The impact of the MOI prior distribution on the posterior predictive distribution of the percentage of blood samples with one or more purely sensitive SNPs in each of the 6000 replicate datasets (datasets are summarised by histograms, which are colour coded by the MOI prior distribution). N. Binomial stands for negative Binomial. The percentage of blood samples with one or more purely sensitive SNPs in the real data is indicated by the black vertical line. The p-value is equal to the fraction (expressed as a decimal number) of replicate datasets that had a higher percentage of blood samples with one or more purely sensitive SNPs than the real data. A p-value that is neither close to zero or one is indicative of model fit with respect to the percentage of blood samples with one or more purely sensitive SNPs.

3.3 Impact of prior assumptions on the genotype frequency estimates

The test statistics (see Section 3.3) are useful indicators of model fit. However, due to the identity function (see Additional file 1), it is difficult to use test statistics to directly summarise fit with respect to genotype frequencies. The results from the analyses under different prior assumptions were therefore compared. It was encouraging to see that the variation between genotype frequency estimates based on different prior assumptions was small compared with estimates generated by direct counting methods[10], especially when the results based on the two distributions that provided the best fit to the data, the Geometric and negative Binomial, were compared (Figure A2.10). Likewise, the precision of the statistical estimates was more consistent than that of the estimates obtained using the counting methods (Figure A2.10).

3.4 Sensitivity of the genotype frequency estimates to the reported mean MOI

Having decided upon a Geometric prior, in light of the sensitivity of the model to the average MOI, the analyses were repeated using estimates at 95% confidence intervals of the reported MOI. The confidence interval was derived using the reported sample standard deviation and a t-distribution[11]. The estimates from the model differed very little across the average MOI range for a given dataset: mean difference 8.77×10^{-4}, ranging from 2.11×10^{-6} to 8.92×10^{-3} (difference between the 00000 genotype estimate, Kanungu, generated using the mean MOI and the MOI at the upper confidence limit). Only the results based on the mean MOI are therefore reported in the main manuscript. Moreover, in the interest of brevity, only those with frequency greater than 0.03 at at least one site are reported (Table 4, main manuscript), despite all theoretically possible genotypes being considered. The genotype frequency estimates are discussed in the results section of the main manuscript.

[10]Three counting methods were used to estimate frequency: discard missing and mixed 1 (discard all patient blood samples in which one or more mixed SNPs were detected, discard all patient blood samples that were missing data due to study design or failed genotyping outcomes, then calculate the proportions of remaining samples with distinct genotypes); discard missing and mixed 2 (assume data missing at SNPs 51 and 108 are mutant type, discard all patient blood samples in which one or more mixed SNPs were detected, and discard all patient blood samples that were missing data at SNPs 59, 437 and/or 540, then calculate the proportions of remaining samples with distinct genotypes); and prevalence (assume data missing at SNPs 51 and 108 are mutant type, score mixed SNPs as mutant type, discard any samples with missing data at SNPs 59, 437 and/or 540, then calculate the proportions of remaining samples with distinct genotypes). Strictly speaking, prevalence is distinct from frequency; prevalence is an estimate of the proportion of patient blood samples that test positive for a collection of mutations (in this case the quintuple mutant, since all mixed SNPs are scored as mutant); whereas, frequency is an estimate of the proportion of parasite clones in the parasite population that carry a given genetic motif (single allele, haplotype or genotype). Here, however, it is used to approximate frequency, therefore highlight the difference between frequency and prevalence.

[11]The reported MOI and standard deviation were external information provided by Francis D et al., J. Infect. Dis. 2006, 193:97886.

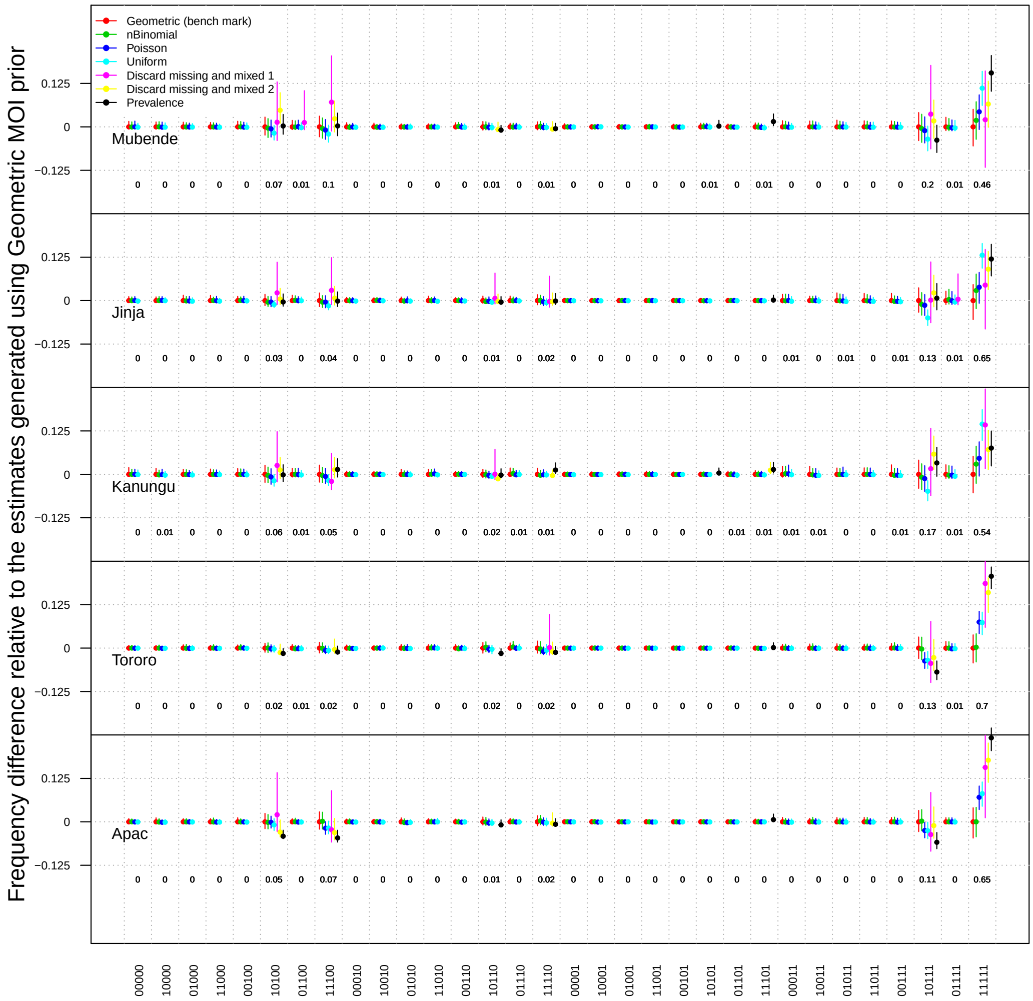

Figure A2. 10: Sensitivity of the geneotype frequency estimates (points) and the 95% credible/confidence intervals (vertical lines) to the MOI prior compared with conventional counting methods. Genotype frequencies are estimates of the proportions of parasite clones in the parasite population that carry genotypes 00000 to 11111, where '1' represents the resistant marker and '0' represents the sensitive marker. The *Pfdhfr* and *Pfdhps* SNPs are ordered as follows: 51, 59, 108 (*Pfdhfr*), 437, 540 (*Pfdhps*). For example, '10111' denotes a quadruple mutant genotype, with a wild type marker in codon 59, *Pfdhfr*. To facilitate comparison, the difference between the estimate generated under the Geometric prior (annotated) and alternative methods (color coded) is plotted. The differences for Geometric estimates are zero since the geometric estimates are the bench marks.

3.5 Insight into the accuracy of analysis of the results based on the field data

The analysis of the simulated data, which mimicked the field data, provided insight into the accuracy of analysis of the field data. In terms of accuracy, precision and coverage, the results suggest that the statistical method is superior to direct counting methods[12] (Figure A2.11).

[12] Again, three counting methods were used to estimate frequency: discard missing and mixed 1 (discard all patient blood samples in which one or more mixed SNPs were detected, discard all patient blood samples that were missing data due to study design or failed genotyping outcomes, then calculate the proportions of remaining samples with distinct genotypes); discard missing and mixed 2 (assume data missing at SNPs 51 and 108 are mutant type, discard all patient blood samples in which one or more mixed SNPs were detected, and discard all patient blood samples that were missing data at SNPs 59, 437 and/or 540, then calculate the proportions of remaining samples with distinct genotypes); and prevalence (assume data missing at SNPs 51 and 108 are mutant type, score mixed SNPs as mutant type, discard any samples with missing data at SNPs 59, 437 and/or 540, then calculate the proportions of remaining samples with distinct genotypes).

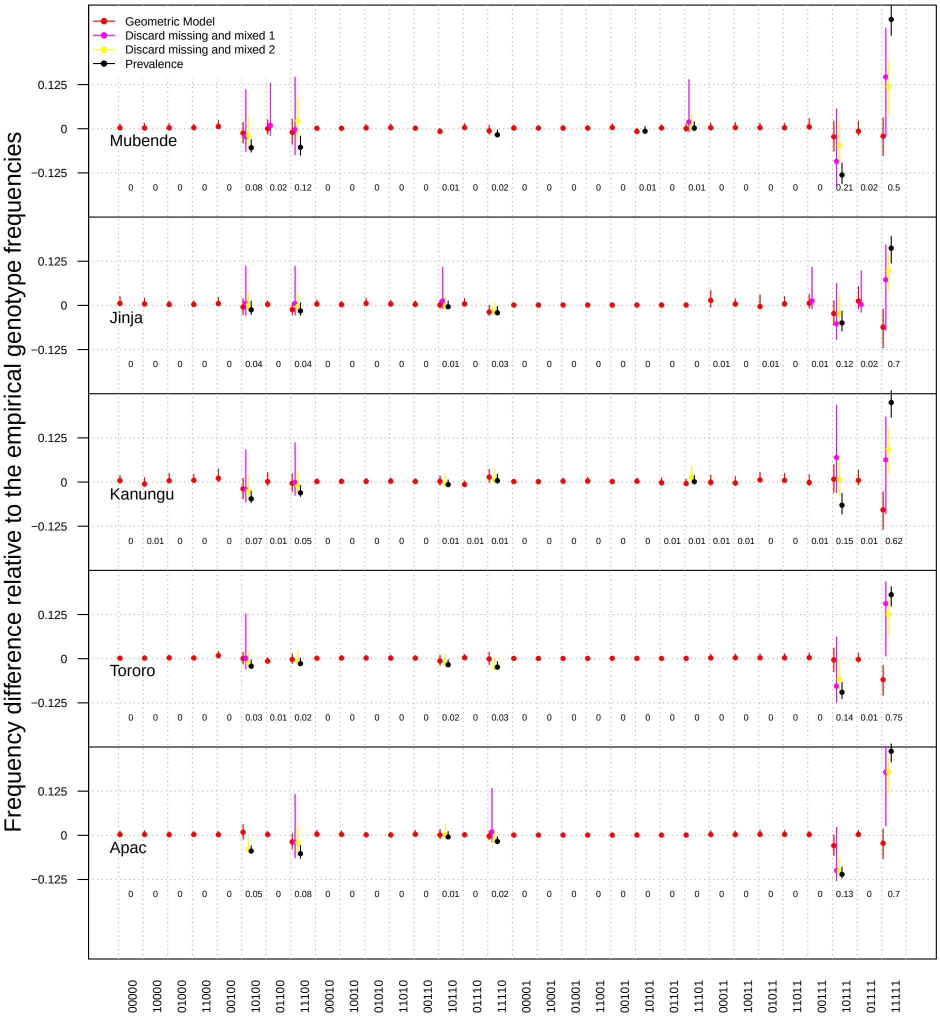

Figure A2.11: Genotype frequency estimates (points) and the 95% credible/confidence intervals (vertical lines), where calcuable, estimated from data simulated such that it resembled the field data. The difference between the estimate and the empirical frequency is plotted. In other words, an estimate located on the zero horizontal is perfect, estimates that have vertical lines that cross the zero horizontal have credible/confidence intervals that span the empirical frequency. Genotypes are indicated by sequences of zeros and ones, where '1' represents the resistant marker and '0' represents the sensitive marker. The *Pfdhfr* and *Pfdhps* SNPs are ordered as follows: 51, 59, 108 (*Pfdhfr*), 437, 540 (*Pfdhps*). For example, '10111' denotes a quadruple mutant genotype, with a wild type marker in codon 59, *Pfdhfr*.
